# Reduced snow cover at the alpine treeline: resistance and recovery of saplings

**DOI:** 10.1111/nph.70926

**Published:** 2026-02-03

**Authors:** Katline Charra‐Vaskou, Guillaume Charrier, Andrea Ganthaler, Thierry Améglio, Stefan Mayr

**Affiliations:** ^1^ Department of Botany University of Innsbruck 6020 Innsbruck Austria; ^2^ INRAE, PIAF Université Clermont‐Auvergne 63000 Clermont‐Ferrand France; ^3^ CNRS, GEOLAB Université Clermont Auvergne 63000 Clermont‐Ferrand France; ^4^ Université Clermont‐Auvergne VetAgro Sup 63370 Lempdes France

**Keywords:** growth, hydraulic conductivity, living cells mortality, nonstructural carbohydrates, resilience, snow removal experiment, stem diameter variation, survival

## Abstract

- At high elevations, tree saplings and shrubs are usually protected by mid‐winter snow cover, although climate change is expected to extend the snow‐free (SF) period. Exposure to winter drought, freeze–thaw events and freezing temperatures will therefore increase, inducing damages to the hydraulic system and to living cells, resulting in reduced growth and increased mortality.
- A snow removal experiment was carried out at 1700 m. above sea level on saplings of five different species (*Acer pseudoplatanus*, *Juniperus communis*, *Larix decidua*, *Picea abies* and *Sorbus aucuparia*). Stem diameter was continuously monitored and compared with spring hydraulic conductivity (PLC_spring_), living cell mortality (PLD_spring_), nonstructural carbohydrates (NSCs), growth and survival rates.
- Under SF conditions, saplings had higher PLC_spring_ and higher PLD_spring_, and thus experienced greater winter dehydration, resulting in lower growth compared with snow‐covered saplings. Summer mortality was strongly correlated with PLC_spring_ and PLD_spring_. These two key ecophysiological parameters predicted the risk of mortality in all species, whereas only PLD_spring_ reduced growth.
- By monitoring stem diameter during winter, we have defined indices to quantify resistance and recovery of woody plants under increased frost pressure. Recovery strategies such as resprouting or embolism repair were critical for survival, highlighting the potential vulnerability of saplings to climate change at high elevations.

## Introduction

Freezing stress is among the most important factors setting the distribution of woody plants at high latitudes and elevations (Sakai & Larcher, 1987; Baranger *et al*., In Press). Although species are currently adapted to their highest observed location, global change is likely to re‐shuffle their adaptive strategies facing climatic stress. As predicted by the IPCC, global change will not simply release frost pressure from constrained environments, but is likely to increase the frequency of extreme climatic events (IPCC, 2012). Due to an increase in mid‐winter thawing events and a decrease in winter solid precipitations, snow cover would become thinner (Sturm *et al*., 1997) and last for a shorter duration (Klein *et al*., 2016).

Snow cover acts as a thermal insulator, protecting plants from extreme air temperatures and reducing thermal fluctuations in the soil and plant tissues (Palm & Tveitereid, 1979; Zhang, 2005; Cai & Li, 2024). This insulation can prevent frost damage and winter desiccation and limit the number of freeze–thaw cycles, especially for small stature plants. Reduction in snow cover would therefore increase plant exposure to frost (lower minimum temperature and higher frequency of freeze–thaw events (FTEs); Groffman *et al*., 2001; Wipf & Rixen, 2010), although the growing season is longer (Wipf & Rixen, 2010). Paradoxically, frost thus is an emerging risk under climate change, as plants may lose protection from snow cover despite overall favorable warmer temperatures (Briceño *et al*., 2014; Saarinen *et al*., 2016).

Taller growth forms (i.e. trees) are more exposed to frost stress whereas smaller growth forms (i.e. shrubs) may be able to avoid it, as FTEs, critically low temperatures and rapid temperature changes are reduced when they are covered by snow. Shrubs may also grow in sheltered microhabitats or favor milder microclimatic conditions (Bannister *et al*., 2005; Ganthaler & Mayr, 2021). At higher elevations (alpine and boreal biomes), shrubs are therefore less exposed to winter temperature stress than tall trees (Körner, 2012). For example, > 100 FTE per winter are frequently observed in south‐exposed distal twigs of pine trees at 2000 m above sea level (asl; Mayr *et al*., 2006a, 2006b), whereas rhododendron shrubs only suffered 40 FTE during winter at a similar elevation (Francon *et al*., 2018). Plant size is therefore a critical trait in explaining frost exposure (Olson *et al*., 2018), but dramatic changes can occur with reductions in snowfall and the resulting changes in height and duration of snow cover.

In the absence of snow cover, lower minimum absolute temperatures and faster temperature changes lead to increased cell damage (especially in normally protected shrubs; Wheeler *et al*., 2014; Charrier *et al*., 2015). Frost damage to living cells is indeed observed when frost tolerance is exceeded (freeze‐induced dehydration) or when temperature changes are too rapid for the cell to maintain its integrity (intracellular ice formation or rapid water influx during thawing; Arora, 2018). Although the physiological limits of frost tolerance are usually reached in mid‐winter (Larcher & Mair, 1968), differences can be observed depending on snow depth (Briceño *et al*., 2014; Palacio *et al*., 2015). Shrubs under snow cover are indeed protected from rapid temperature changes (Saarinen & Lundell, 2010; Deslauriers *et al*., 2021). Contrastingly, the absence of snow cover can damage all organs, but the most critical damage is observed when the root system is affected (Ambroise *et al*., 2020). Indeed, belowground damage affects the capacity of the root system to maintain its hydraulic function, which is highly critical for growth resumption.

Frost tolerance of living cells is related to the dynamics of soluble carbohydrates and polyols (Charrier *et al*., 2013a, 2013b; Baffoin *et al*., 2021; Deslauriers *et al*., 2021). During cold acclimation, starch reserves are hydrolyzed into soluble carbohydrates and the reverse occurs during cold deacclimation (Sakai, 1966; Améglio *et al*., 2004; Charrier *et al*., 2018). The accumulation of soluble carbohydrates in living tissues enhances the probability of extracellular ice formation by decreasing the freezing point of intracellular compartments (Sauter, 1988; Lee & Thomashow, 2012). Carbohydrates also protect the cells by stabilizing membranes and proteins under freeze‐induced dehydration (Imanishi *et al*., 1998; Charrier *et al*., 2013a, 2013b). At growth resumption, starch reserves are again hydrolyzed to supply the formation of new tissues.

Frost is also critical for the hydraulic compartment, as FTE induce gas embolism in the conducting elements of the plant xylem. According to the ‘thaw‐expansion hypothesis’, air bubbles, which are entrapped in the ice lattice when xylem sap turns into ice, expand on thawing and embolize the whole xylem conduit (Hacke & Sperry, 2001; Charrier *et al*., 2014; Charra‐Vaskou *et al*., 2016, 2023). Wider conduits, containing higher volumes of dissolved gas, thus form bigger bubbles at freezing, which would expand at lower tension than smaller ones, according to Jurin's law, making these conduits more vulnerable (Cruiziat *et al*., 2002; Pittermann & Sperry, 2003, 2006). Xylem conduit dimension has thus been related to xylem's safety to freezing damage (Davis *et al*., 1999), and accordingly, narrower conduits are more represented in frost‐exposed environment (Zanne *et al*., 2018). Conifer species with narrow tracheids are more resistant to freezing‐induced embolism, unless they are exposed to high tensions (Mayr *et al*., 2007). As a consequence, avoidance of critical tensions is another important strategy (Pittermann & Sperry, 2006). The latter might be favored by low stomatal leakiness and thicker cuticle, efficient water uptake or internal water storage. For instance, frozen xylem sections can provide large pools of stored water, which are released when the ice melts (Mayr & Charra‐Vaskou, 2007).

Higher number of daily FTE would exacerbate winter drought and xylem embolism (Tranquillini, 1982; Mayr & Améglio, 2016). The access to soil water is very limited when the soil, the root system or xylem sections are frozen and do not allow water transport for months (Mayr *et al*., 2014, 2019). Meanwhile, above the snow, dehydration is enhanced when branches are overheated (high radiation during sunny winter periods, amplified by albedo) and air is dry (low relative humidity; Charrier *et al*., 2017). Even deciduous plants, which are less prone to water losses by shedding leaves, can suffer from drought stress through peridermal transpiration (Burghardt & Riederer, 2003).

Although several studies have reported on the effects of lack of snow cover on the survival and growth of different species (Frey, 1983; Drescher & Thomas, 2013; Domisch *et al*., 2018), very few have examined neither physiological parameters, such as cell damage (Comerford *et al*., 2013) or NCS reserves (Domisch *et al*., 2019), nor anatomical traits (Power *et al*., 2024). Other physiological indicators that have been used to assess winter stress and recovery include measurements of frost tolerance, tissue water content, water potential and photosynthetic activity in evergreen species. Although the hydraulic compartment is a key factor explaining plant distribution at high elevation (Mayr *et al*., 2003, 2014; Charrier *et al*., 2013a, 2013b), hydraulic failure has not been measured under these conditions.

In this context, stress resistance and recovery will define which shrub and tree species will be adapted to lower snow depth and shorter snow cover duration. The resistance refers to the ability of saplings to survive and adapt to stressful environmental conditions. Species with high resistance to dehydration, to extremely low temperature and to FTE‐induced embolism are likely to better withstand these conditions. Contrarily to DeSoto *et al*. (2020) and Lloret *et al*. (2024) who defined resilience on a comparison of observed variables to the reference state on a large spatiotemporal scale, recovery mechanisms, that is the ability of saplings to restore their physiological state after a period of stress, can be achieved through restoring water balance, resorption of embolism and/or secondary growth during a shorter temporal scale. Physiological adaptations that enable trees to survive, restart growth or produce new leaves and stems after a period of stress will select species adapted to future climatic conditions.

To assess stress resistance and recovery, an efficient way to measure cell damage, growth and water status nondestructively in trees is the use of microdendrometers (Zweifel & Häsler, 2000). Microdendrometers are commonly used to monitor growth and drought stress in woody plants (Reineke, 1932; Daubenmire, 1945; Deslauriers *et al*., 2007; De Swaef *et al*., 2015; Dobbert *et al*., 2022) as well as freezing stress (Charra‐Vaskou *et al*., 2016; Charrier *et al*., 2017). Upon freezing, a sharp decrease in diameter is observed, which does not completely restore in damaged plants (Améglio *et al*., 2001). While reversible minimum diameter reached corresponds to drought stress intensity (Charrier *et al*., 2017; Lamacque *et al*., 2020), the proportion of irreversible diameter shrinkage is an index of freeze‐induced damage to living cells (Lintunen *et al*., 2015; Lamacque *et al*., 2020). In the lavender shrub, Lamacque *et al*. have related the percent loss of diameter (PLD) and the percent loss of rehydration capacity (PLRC) with hydraulic damages and vitality (Lamacque *et al*., 2020, 2022).

For shrubs, snow manipulation experiments designed to simulate the expected advance of melt out dates in a warmer climate indicated a strong control of snow cover duration on phenology and growth by modulating growing season length, soil temperature, nutrients availability and frost occurrence (Wipf & Rixen, 2010; Gerdol *et al*., 2013; Rixen *et al*., 2022). Snow manipulation experiments are an efficient tool to analyze the complexity of the interactions between snow cover, frost, water availability and vegetation. However, physiological studies in relation to snow cover duration are few. *Vaccinium vitis‐idaea* shrubs exhibited similar frost tolerance in winter under different snow conditions, but plants lacking snow cover suffered from water loss and increased photoinhibition (Taulavuori *et al*., 2011). However, it was also found that alpine *Ericaceae* shrubs under low snow cover tended to be more frost‐resistant and had higher sugar concentrations than those covered by thick snow layers (Saarinen *et al*., 2016).

As predicted by the IPCC, global change will lead to shorter and thinner winter snow cover, impacting trees and shrubs' survival and growth, as well as regeneration. As the current knowledge gap regarding complex winter stress patterns is a key issue, this study aimed to determine the ecophysiological parameters involved. To assess the effect of snow cover on hydraulic integrity, living cell mortality, survival and growth, we conducted a snow removal experiment at high elevation (1700 m asl) on saplings of five angiosperm and conifer shrub and tree species and monitored key ecophysiological parameters. We hypothesized that (1) lack of snow cover will induce frost damage on living cells and hydraulic compartments, (2) different woody species will react according to their specific physiological thresholds and (3) survival to snow‐free (SF) winter conditions will depend on the amount of both hydraulic and cell damage and on the ability to mobilize carbohydrates to supply re‐growth.

## Materials and Methods

### Plant material and study site

In spring 2021, 100 saplings (2–4 yr old, *c*. 50 cm tall, 1–1.5 cm in diameter at the base of the main stem) of 2 angiosperm species (*Sorbus aucuparia* L. and *Acer pseudoplatanus* L.) as well as 3 conifer species (*Larix decidua* Mill., *Picea abies* (L.) H. Karst and *Juniperus communis* L.) from local seed sources were bought from a commercial tree nursery, grown for the summer in the Institute of Botany's nursery in Innsbruck to avoid summer stress at the high elevation site and planted in October 2021. The experimental site was located in Praxmar, Sellrain (47.1550 N; 11.1350 E; 1650 m asl), in the Tyrolean Central Alps (Austria) on a south‐east exposed slope. Two sets of 10 trees per species were randomly placed in two plots. From November 1 to March 25 (start day of snow melt, i.e. defined as the ‘winter period’), snow was removed by hand from the SF plot within 1 d after each snowfall. In the second plot, trees were kept covered by natural snowfall and additional snow from the first plot (snow‐covered (SC) plot). These species were chosen to analyze angiosperm (2) vs conifer (3) species, deciduous (3) vs evergreen (2) as well as tall (4) vs shrub (1) forms. Both plots were free of snow from April 6.

On both plots, continuous measurements (soil water potential, air, soil and stem temperatures and stem diameter variation) were performed from October 2021 to October 2022. In addition, physiological measurements were made in spring (May 2022, just after snowmelt and before the start of growth) and in summer (August 2022, end of the growing season).

### Continuous measurements

Soil temperature and soil water potential were measured at a depth of 10 cm using MicroLog SP3 (Delmhorst gypsum blocks; EMS, Czech Republic) on each experimental plot (*n* = 3 per plot) at an interval of one or two measurements per hour throughout the experiment. The measuring range of the water potential sensors was 0 to −1.44 MPa. A weather station was installed at 2 m high on one side of the plot, measuring every 30 min: air temperature, relative humidity, wind speed and photosynthetically active radiation (Capt‐connect, France). Snow depth along a graduated wooden ruler and phenological stages (budbreak and leaf fall) were monitored on both plots using a timelapse camera (TLC200 Pro; Brinno, the Netherlands). A picture was taken every day at noon.

Stem diameter was monitored using linear variable differential transducer (LVDT) sensors on 4 trees per species and treatment (*n* = 39, corresponding to the number of channels available on the data loggers). The sensors were installed on the main stem of the trees at 10 cm above the ground, between 20 October and 25 October 2021 for the whole duration of the experiment. In addition, the temperature of the stems was measured using thermocouples (type T) installed within the bark of the stems, 5 cm away from the LVDT sensor, on 3 trees per species and treatment (*n* = 30). The LVDTs and thermocouples were connected to two data loggers (CR 1000; Campbell, Logan, UT, USA) which took three measurements per minute and recorded the 1‐min average. Initial stem diameter values were set on 23 November 2021. The predawn stem diameter (*c*. 3:00 h) was chosen as the representative value for each day. Stem freeze–thaw cycles were estimated when temperatures increased from below −2°C to above +2°C and *vice versa*.

### Winter measurements

The percent loss of diameter in winter (PLD_winter_) was calculated using the change in diameter between 23 November 2021 and 1 February 2022 (Ø_Feb_). This date was representative of the maximum stress intensity experienced by the stems during the extended mid‐winter frozen period. PLD_winter_ was the normalized value of Ø_Feb_ by stem diameter manually measured in May 2024 and corrected by the change (*i.e*. growth and stress accumulation) observed during the monitoring period (Ø_Ref_). PLD_winter_ was thus calculated as:

(Eqn 1) $${\mathrm{PLD}}_{\text{winter}}=100*\frac{{ø}_{\mathrm{Feb}}}{{ø}_{\mathrm{Ref}}}$$

### Spring measurements

Spring xylem hydraulic damages were assessed by measuring percent of hydraulic loss of conductivity (PLC_spring_) by high‐resolution computed tomography (HRCT). Samples were collected on May 10 on three to six replicates per species and conditions. PLC_spring_ was measured on lateral branches (most species) or on the terminal section of the main stem (*Acer sp*). Samples of *c*. 10 cm (conifers) or 20 cm (angiosperms) in length were harvested, wrapped in plastic bags and transported to the laboratory. The stems were then recut at least three times on each end under water to reach 5‐cm long samples. Sample ends were sealed with warm liquid Parafin to block all open conduits and the entire stem was wrapped in Parafilm (Alcan, QC, Canada) to prevent dehydration. Embolism measurements were performed at the PIAF laboratory (INRAE Clermont‐Ferrand, France) using a bench X‐ray microtomograph (Nanotom 180 XS; GE, Germany). This method is based on the local X‐ray absorption behavior of the sample mainly according to the local density. It provides direct observation of the internal structure of an intact sample without surface or cutting preparation (Charra‐Vaskou *et al*., 2012; acquired during the 360° rotation of the sample). The 3D reconstruction was performed using the Phoening datosx 2 software (General Electric, Wunstorf, Germany) and provided a spatial resolution of 3.8 μm per voxel. One representative transverse cross‐section of the 3D tomographic volume was extracted from the center of the volume for each scan. Air‐filled vessels were highly contrasted with the surrounding tissue and converted to binary images using the ImageJ software (http://rsb.info.nih.gov/ij). The diameter and area of each individual air‐filled conduit was extracted and measured in the whole cross‐section, discarding lumina with areas smaller than 10 μm^2^ (i.e. *c*. 3 voxels). At the end of the experiments, a final reference scan, with all conduits filled with air was then recorded. The total number of conduits (*N*_tot_) and the total area (*A*_tot_) were measured in the cross‐section. In angiosperm species, although hydraulic conductivity is mainly influenced by the cross‐sectional area of the conduits, individual conduits are easily identifiable. In this study, the angiosperm species were diffuse‐porous with a homogeneous distribution of conduits diameters, so percent loss of conductivity of a stem could be computed as the ratio of air‐filled conduits number to the total number of conduits. In conifers, the diameter of individual conduits was closer to the image resolution so the ratio of the area of air‐filled conduits to the total area of conduits was a good proxy for percent loss of conductivity. In *Betula sp*, there was no significant difference between the two computations (Charra‐Vaskou *et al*., 2023).

For angiosperm samples, the PLC_spring_ was calculated as:

(Eqn 2) $${\mathrm{PLC}}_{\text{spring}}=100*\frac{N_{\mathrm{afc}}}{N_{\mathrm{tot}}}$$

with *N*_afc_ corresponding to the total number of air‐filled conduits and *N*_tot_ the total number of conduits.

For conifer samples, the PLC_spring_ was calculated as:

(Eqn 3) $${\mathrm{PLC}}_{\text{spring}}=100*\frac{A_{\mathrm{afc}}}{A_{\mathrm{tot}}}$$

with *A*_afc_ corresponding to the area of air‐filled conduits and *A*_tot_ the total area of the xylem.

In addition to PLC_spring_ measurement by HRCT, safranine staining was performed on samples collected on the same day. Samples were sealed in a hydraulic system (modified from Sperry *et al*., 1988) connected to a reservoir filled with 0.1% (wt/vol) filtered (0.22 μm) safranin solution. The pressure was set to 5 kPa, and the samples remained connected until the outflow was deeply red stained. After a drying period of 30 min at room temperature, cross‐sections were made from the middle part of the stained stem sections with a slide microtome (Schlittenmikrotom G.S.L. 1, Schenkung Dapples, Switzerland) and analyzed with a light microscope (Olympus BX41; Olympus, Vienna, Austria) connected to a digital camera (ProgRes CT3; Jenoptik, Jena, Germany).

Percent loss of diameter in spring (PLD_spring_) was determined from LVDT values measured on 1 April 2022 (Ø_Apr_). This date was chosen because the snow had already melted (favorable temperatures and hydration conditions) and the soil water potential had been above −0.2 MPa for 13 d, but growth had not yet started. PLD_spring_ was the normalized value of Ø_Apr_ by Ø_Ref_:

(Eqn 4) $${\mathrm{PLD}}_{\text{spring}}=100*\frac{{ø}_{\mathrm{Apr}}}{{ø}_{\mathrm{Ref}}}$$

Spring relative electrolyte leakage (REL_spring_) was measured on samples harvested on May 10 in four to six replicates for all species and conditions. This method is classically used to determine cell damages induced by stress (Zhang *et al*., 1993).

About 10 slices of stem (1 mm thick) were cut with a razor blade, placed in test tubes containing 1.5 ml of distilled deionized water. Tubes were placed in a shaker with thermoregulation (ThermoMixer C; Eppendorf, Hambourg, Germany) and shaken for 1 h at 25°C. The conductivity of the solution (C_1_) was measured with a conductivity meter (LAQUAtwin EC‐22; Horiba, Kyoto, Japan). Then the tubes were again shaken for 30 min at 100°C followed by another 30‐min shaking at 25°C. After cooling down to room temperature, a second conductivity measurement (C_2_) was performed. The REL_spring_ was thus calculated as:

(Eqn 5) $${\mathrm{REL}}_{\text{spring}}=100*\frac{C1}{C2}$$

The content of nonstructural carbohydrates in the spring (Starch_spring_, Sucrose_spring_, Glucose_spring_ and Fructose_spring_) was performed on samples collected on 10 May 2022 on lateral branches or on the terminal section of the main stem (mainly Acer). Lyophilized samples (m > 2 g) were ground into a powder, which was used (50 mg) for extraction of soluble carbohydrates. It was soaked with 1 ml of mannitol (5 g l^−1^) in ethanol 80%, shaken at 80°C for 30 min and then centrifuged at 15 775 ***g*** for 10 min (SR2000; Prolabo, Ouled Fayet, Algeria). The supernatant was filtered in a cartridge containing AGX‐1 anion‐exchange resin (150 μl), polyvinylpolypyrrolidone (100 μl) and activated carbon (200 μl). The solid was melted three more times with ethanol 80% (1 ml), ethanol 50% (0.5 ml) and ethanol 80% (0.5 ml), before the cartridge was rinsed with ethanol 80% (1 ml). The liquid and solid fractions were conditioned separately and SpeedVac‐dried for soluble carbohydrates and starch analysis, respectively. For carbohydrate analysis, samples were diluted in 0.5 ml of water and separated on an Aminex‐HPX87C Column with a refractometer (R12000; Sopares). Based on retention index, to measure spring starch content, the solid was melted with NaOH 0.02 N and autoclaved (2 h, 120°C, 1 bar). The samples were then incubated with amyloglucosidase (1h30mn, 52°C) in a microplate well, each well containing 12 μl ATP (5 × 10^−4^ mol l^−1^), 12 μl NADP (1.4 × 10^−4^ mol l^−1^), 60 μl triethanolamine buffer (triethanolamine 0.48 mol.l^−1^, magnesium sulfate 1 × 10^−2^ mol l^−1^, pH = 7.6), 96 μl water and 12 μl of sample supernatant. A spectrophotometric measurement at 340 nm (Power Wave 200; BioTek Instruments, Winooski, VT, USA) was performed as a blank before incubation with 10 μl of hexokinase/glucose‐6‐phosphate dehydrogenase (EC 1.1.1.49) for 40 min with shaking, followed by another absorbance measurement.

### Summer measurements

Summer relative diameter growth (RDG) was determined as follows:

(Eqn 6) $$\mathrm{RDG}=100*\left({\mathrm{PLD}}_{\text{autumn}}-{\mathrm{PLD}}_{\text{spring}}\right)$$

where PLD_autumn_ (percent loss of diameter in autumn) was calculated using LVDT values measured on 1 October 2022 (Ø_Oct_) and according to:

(Eqn 7) $${\mathrm{PLD}}_{\text{autumn}}=100*\frac{{ø}_{\mathrm{Oct}}}{{ø}_{\mathrm{Ref}}}$$

All stem diameter values were averaged per species and per treatment (*n* = 3 or 4 for each treatment and species).

The summer mortality index (MI_summer_) was analyzed on 29 July 2022. According to the general visual state of the saplings, each individual was scored from 1 (healthy) to 5 (dead) as follows: 1: for green and healthy trees; 2: when one branch was brown or apparently dead; 3: when at least two branches were brown or apparently dead with at least one branch green and alive; 4: when severe damage was observed on the entire aboveground part but with resprouting from the root system; and 5: when the tree appeared completely dead (completely brown or visually dead).

### Statistical analyses

The effect of snow cover was assessed on all measured parameters by using the nonparametric Kruskal–Wallis rank sum test (*kruskal.test* function in R; Rstudio, v.4.3.2; Posit, PBC, MA, USA) as the data were, in most cases, not normally distributed. The tests were performed on the full dataset or on different subsets corresponding to species and/or functional groups (Supporting Information Dataset S1). The correlations between monitored variables were assessed through Pearson correlation (*t.test* function in R). We assessed the potential of physiological variables to predict MI_summer_ and RDG by comparing two approaches. The first approach used only qualitative state variables, such as conditions, species, growth form, leaf lifespan or wood anatomy, to predict mortality and growth indexes. The second approach used quantitative physiological variables (PLD_winter_, PLC_spring_, PLD_spring_, REL_spring_, Starch_spring_ and carbohydrates contents) as predictive variables.

Different linear models were tested (*glm* function in R), and the best was selected according to the statistical significance for each factor and according to several indexes: root mean squared error (RMSE), adjusted R‐squared (Adj.R^2^) and corrected Akaike index criterion (AICc; Akaike, 1974; Hurvich & Tsai, 1995).

(Eqn 8) $$\text{RMSE}=\sqrt{\frac{\sum_{i=1}^{n}{\left({\hat{y}}_{i}-y_{i}\right)}^{2}}{n}}$$

(Eqn 9) $$\mathrm{Adj}.R^{2}=1-\frac{n\cdot \left(n-1\right)\cdot {\text{RMSE}}^{2}}{\left(n-k\right)\cdot \sum_{i=1}^{n}{\left(\;y_{i}-\bar{y}\right)}^{2}}$$

(Eqn 10) $$\text{AICc}=2n\left[\log\left(\text{RMSE}\right)+\frac{k}{n-k-1}\right]$$

with $\hat{y}$_*i*_ the values simulated by the model for an individual *i*, *y*_*i*_ the observed values for an individual *i*, *k* the number of parameters, *n* the number of observations and $\bar{y}$ their mean.

The robustness of the model was assessed based on Prediction RMSE (RMSEP) computed from one‐leave‐out cross validation. The calibration was performed on all but one observation and the predicted value $\hat{y}$_i_ estimated on that remaining observation.

## Results

### Stress intensity depending on snow cover

**Fig. 1:**
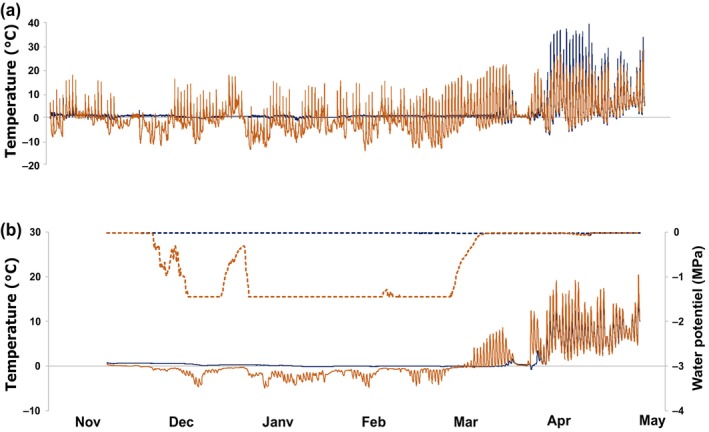
Time course of air (°C) and soil temperature (°C; a) and soil water potential (MPa; b) from 5 November 2021 (air temperature) or 23 November 2021 (soil temperature and water potential) to 10 May 2022. Conditions in snow‐covered and snow‐free plots are represented by the blue and orange lines, respectively.

The absence of snow cover in winter resulted in very different environmental conditions in the SF compared with the SC plot (Fig. 1). In the SC plot, the saplings were completely covered by snow from the beginning of November 2021 until the end of March 2022, whereas the saplings in the SF plot were never covered by snow for > 2 d and the entire aboveground part of saplings was exposed to the air temperature shown in Fig. 1. The first snow fell on 1 November 2021 and the last snow melted between 25 March and 6 April 2022. While in the SC plot, leaf fall could not be monitored due to already present snow cover, and in the SF plot, leaf fall occurred shortly after, that is before November 15.

In the SC plot, the microclimatic conditions remained relatively stable, especially compared with the SF plot. Soil temperatures stayed between −0.4 and 0.7°C (SC) compared with −4.9°C to 7.2°C (SF) at 5 cm depth (data not shown) and between −0.2 and 0.8°C (SC) compared with −3.6°C to 4.2°C (SF) at 10 cm depth (Fig. 1). Stem temperature was also buffered in the SC plot (between −1.8 and 8.2°C) compared with the SF plot (from −14.0°C to 21.6°C). Consequently, saplings in the SC plot experienced no freeze–thaw cycles before and only 10 after snowmelt (between March 25 and April 28), whereas SF trees accumulated 53 freeze–thaw cycles during the winter.

Winter soil water potential was stable in the SC plot, remaining between −0.02 and −0.03 MPa. In the SF plot, winter soil water potential remained above −0.05 MPa before December 7 and after March 21. When the soil froze, values down to −1.5 MPa were recorded (Fig. S1).

### Winter stem diameter shrinkage

**Fig. 2:**
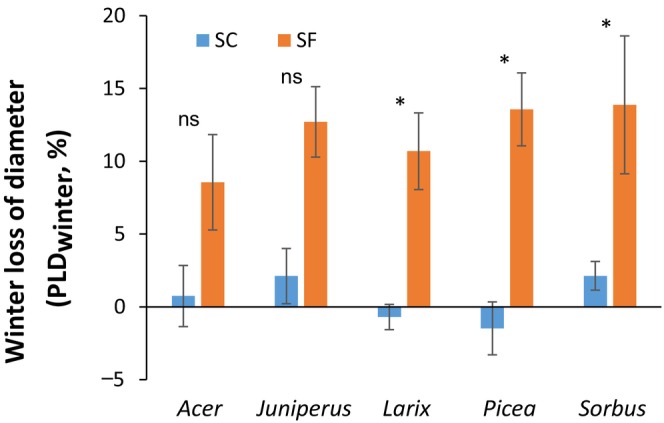
Percent loss of diameter in mid‐winter (PLD_winter_; 1 February 2022) of saplings of the five species growing in snow‐covered (SC) and snow‐free (SF) conditions. Kruskal–Wallis test results are indicated by ‘ns’ for not significant (*P* > 0.05) and an asterisk for significant (*P* < 0.05) differences between SF and SC. Bars and whiskers represent the mean and the SE, respectively.

In winter, SF trees showed significantly higher stem diameter shrinkage (percent loss of diameter in mid‐winter, PLD_winter_ from 8.6% for *Acer* to 13.9% for *Sorbus*) than SC trees (PLD_winter_ from −1.5% for *Picea* to 2.1% for *Sorbus*; *P* < 0.001; Fig. 2), as also illustrated in Fig. S1. However, at the species level, the difference in PLD_winter_ was not systematically significant, mainly due to the large inter‐individual variability in SF trees (*P* = 0.157 and 0.275 in *Acer* and *Sorbus*, *P* < 0.034 in the other species).

### Spring key ecophysiological parameters

#### Vascular system, irreversible diameter shrinkage and NSC content

**Fig. 3:**
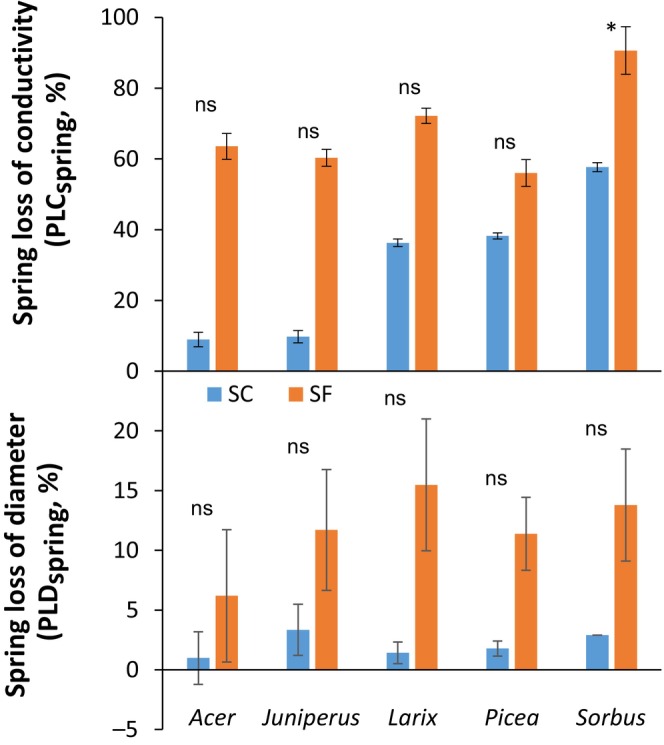
Percent loss of hydraulic conductivity in spring (PLC_spring_) and percent loss of diameter in spring (PLD_spring_) of trees of the five species growing in snow‐covered (SC) and snow‐free (SF) conditions. Kruskal–Wallis test results are indicated by ‘ns’ for not significant (*P* > 0.05) and an asterisk for significant (*P* < 0.05) differences between SF and SC. Bars and whiskers represent the mean and the SE, respectively.

In spring, immediately after snowmelt, higher loss of hydraulic conductivity was observed in SF trees (percent loss of conductivity in spring, PLC_Spring_ of 77.8 ± 3.7%, mean ± SE) compared with SC trees (PLC_Spring_ of 31.3 ± 3.9%, mean ± SE; *P* < 0.001, Fig. 3). A higher value than 80% was observed in three species (PLC_Spring_ of 90.6 ± 2.0%, 86.2 ± 1.1% and 82.4 ± 1.1% for *Sorbus*, *Picea* and *Larix*, respectively), whereas PLC_Spring_ remained lower in the other two species (63.5 ± 13.4% and 54.5 ± 8.0% for *Acer* and *Juniperus*, respectively). Under SC conditions, a high PLC_Spring_ was also observed in *Sorbus* (57.7 ± 0.8%), whereas it remained lower than 50% for *Larix* and *Picea* and even lower than 10% for *Acer* and *Juniperus*. Interestingly, the species that exhibited the lowest PLC_spring_ in SC conditions (*Acer* and *Juniperus*) showed the highest difference in PLC_spring_ between SF and SC conditions.

**Fig. 4:**
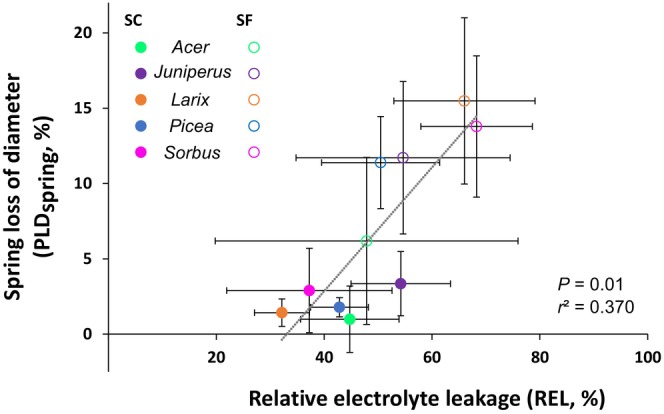
Relative electrolyte leakage in spring (REL_spring_) with percent loss of diameter in spring (PLD_spring_) of snow‐covered (SC) and snow‐free (SF) saplings of the five species. The dotted line indicates the best linear regression.

**Fig. 5:**
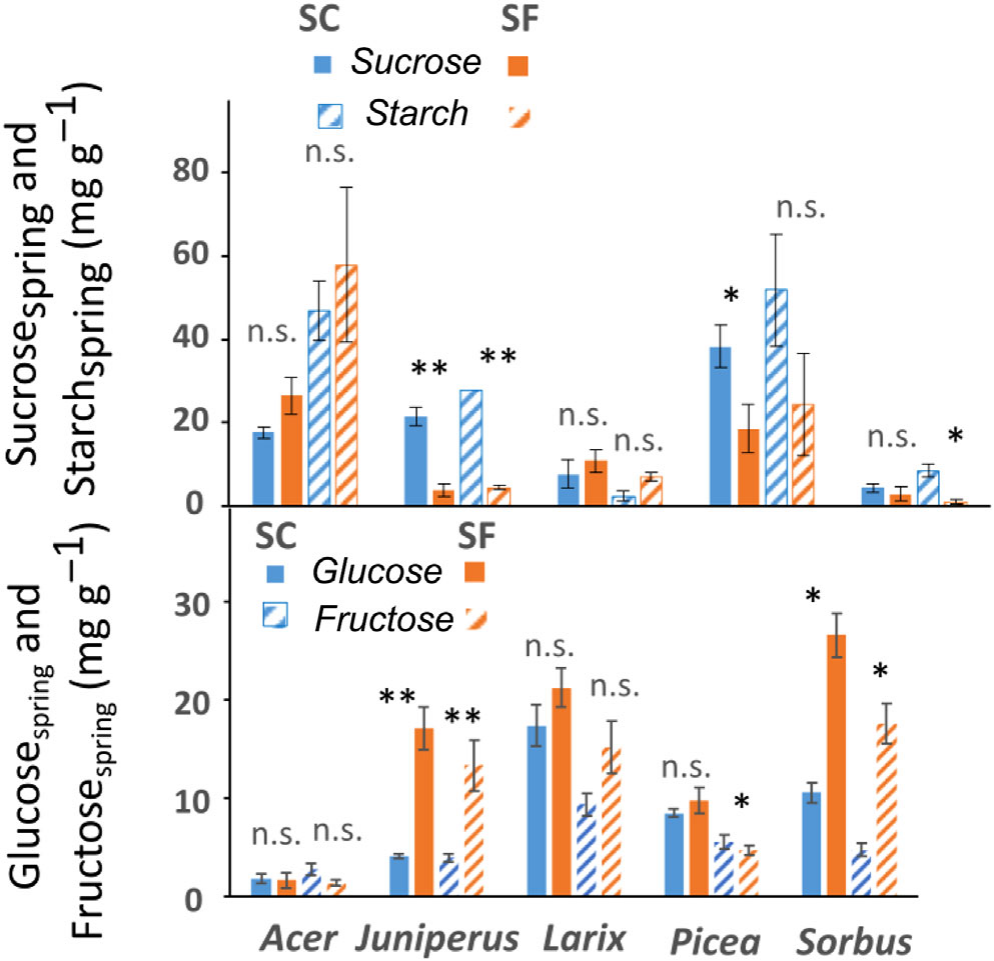
Sucrose_spring_, Starch_spring_, Glucose_spring_ and Fructose_spring_ (corresponding to spring contents in mg g^−1^) of snow‐covered (SC) and snow‐free (SF) saplings of the five species. Kruskal–Wallis test results are indicated by ‘ns’ for not significant (*P* > 0.05) and ‘*’ and ‘**’ for significant (*P* < 0.05 and *P* < 0.01, respectively) differences between SF and SC. Bars and whiskers represent the mean and the SE, respectively.

Saplings under SC conditions showed very low stem diameter shrinkage (PLD_spring_ of 2.1 ± 0.5%, mean ± SE) compared with SF conditions (PLD_spring_ of 11.6 ± 1.5%, mean ± SE; *P* = 0.015, Fig. 3). However, at the species level, only *Larix* and *Picea* showed significant differences between treatments (*P* = 0.034). PLD_spring_ was positively correlated with REL_spring_ regardless of the species (*R* ^2^ = 0.370; *P* = 0.010; Fig. 4). However, two clusters of points represented SF and SC conditions, although the values from *Juniperus* or *Acer* in SC conditions were relatively far from the regression. Similarly, the percentage of visualized cellular mortality in the bark only (to make it comparable between species) in May 2022 was significantly correlated with PLD_spring_ (Fig. S2). Although no significant effect of the treatment was observed on total nonstructural carbohydrates (*P* = 0.975), starch (*P* = 0.062) nor total soluble carbohydrates contents (*P* = 0.279), significant differences were observed for individual compounds. In spring, Glucose_spring_ and Fructose_spring_ were twofold higher under SF conditions (Glucose_spring_ of 15.3 ± 3.9 and 8.5 ± 2.4 mg g^−1^ DM under SF and SC conditions, respectively, *P* = 0.009; and Fructose_spring_ of 10.5 ± 2.8 and 5.3 ± 1.0 mg g^−1^ DM under both conditions, respectively, *P* = 0.024, mean ± SE; Fig. 5). Although being lower under SF conditions, no significant differences were observed for Sucrose_spring_ (*P* = 0.124) or Starch_spring_ (*P* = 0.062).

#### Correlation between key ecophysiological parameters and winter stress intensity

**Fig. 6:**
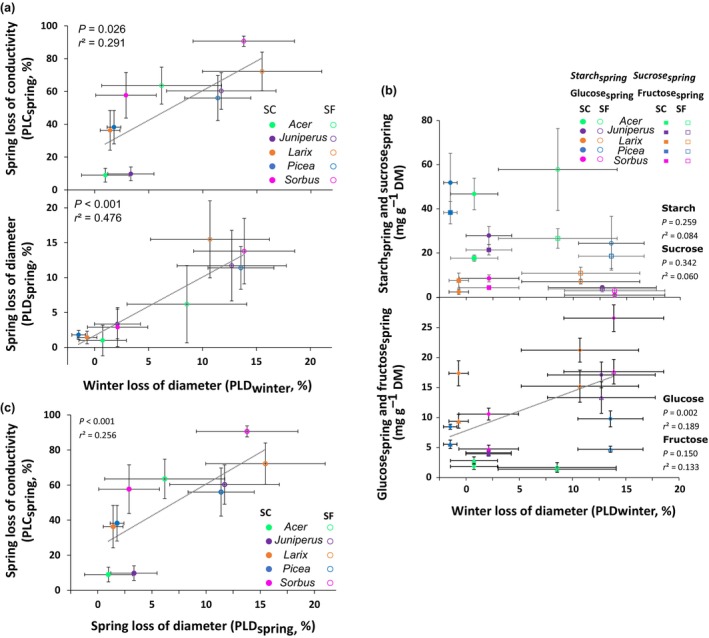
Spring loss of hydraulic conductivity and diameter and sucrose, glucose and fructose contents depending on winter loss of diameter as well as the relation between spring loss of conductivity and spring loss of diamter. (a) Percent loss of hydraulic conductivity in spring (PLC_spring_), percent loss of diameter in spring (PLD_spring_) of snow‐covered (SC) and snow‐free (SF) saplings of the five species depending on winter stem diameter shrinkage (PLD_winter_). Symbols and bars represent means and SEs, respectively. The dotted line indicates the best linear regression. (b) Starch_spring_, Sucrose_spring_, Glucose_spring_ and Fructose_spring_ (corresponding to spring contents in mg g^−1^) of snow‐covered (SC) and snow‐free (SF) saplings of the five species depending on mid‐winter stem diameter shrinkage (PLD_winter_). Symbols and bars represent means and SEs, respectively. The dotted line indicates the best linear regression. (c) Correlation between Spring loss of hydraulic conductivity (PLC_spring_) and Spring loss of diameter (PLD_spring_) of snow‐covered (SC) and snow‐free (SF) saplings of the five species. Full symbols represent trees growing on SC conditions and empty symbols on stressed conditions. Symbols and bars represent means and SEs, respectively. The dotted line indicates the best linear regression.

PLC_spring_ was positively correlated to PLD_winter_ (*R* ^2^ = 0.291; *P* = 0.026; Fig. 6a) although some species did not experience substantial shrinkage under SC conditions. Under SF conditions, high PLC_spring_ (between 56.0 and 90.6%) and PLD_winter_ (between 8.6% and 13.9%) were observed. *Larix* and *Sorbus* showed the highest PLC_spring_ in SF conditions (72.2 ± 11.8% and 90.6 ± 3.2%, respectively). *Picea*, *Acer* and *Juniperus* showed only moderate PLC_spring_ (56.0 ± 13.7%, 63.5 ± 11.2% and 60.3 ± 11.2%, respectively) despite high PLD_winter_ (13.6 ± 1.6%, 8.6 ± 2.1% and 12.7 ± 1.5%, respectively).

PLD_spring_ was positively correlated with PLD_winter_ (*R* ^2^ = 0.476; *P* < 0.001; Fig. 6b). For SC saplings, weak shrinkage was observed in winter (PLD_winter_ from −0.7% to 2.1%) as well as in spring (PLD_spring_ from 1.0% to 3.3%). In SF saplings, the high PLD_winter_ (8.6–13.9%) was not recovered in spring (PLD_spring_ 6.2–13.8%). Among the different carbohydrates, only Glucose_spring_ was correlated with PLD_spring_ (*P* = 0.047; Fig. 6b). In spring, the two physiological parameters PLC_spring_ and PLD_spring_ were positively correlated, and species with the highest PLD_spring_ also had the highest PLC_spring_, regardless of the conditions (Fig. 6c).

### Summer relative diameter growth and summer mortality index

**Fig. 7:**
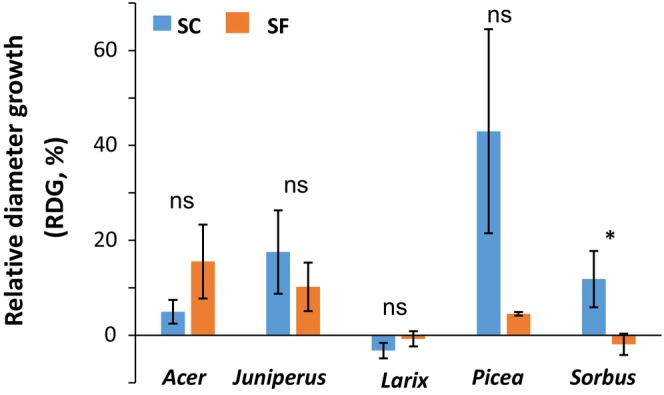
Relative diameter growth (RDG) of saplings of the five species growing in snow‐free (SF) and snow‐covered (SC) conditions. Kruskal–Wallis test results are indicated by ‘ns’ for not significant (*P* > 0.05) and an asterisk for significant (*P* < 0.05) differences between SF and SC. Bars and whiskers represent the mean and the SE, respectively.

Although SC saplings exhibited substantial RDG, SF conditions significantly reduced growth (Fig. 7), especially for *Picea* (RDG of 43.0% and 4.5% in SC and SF conditions, respectively; *P* = 0.480) and *Sorbus* (RDG of 11.9% and −1.9% in SC and SF conditions, respectively; *P* = 0.050). No significant effect of SC conditions on RDG was observed for *Acer* (5.0% and 15.6% in SC and SF conditions, respectively; *P* = 0.480), *Juniperus* (RDG of 17.6% and 10.2% in SC and SF conditions, respectively; *P* = 0.480), and *Larix*, which showed almost no growth (RDG of −3.2% and −0.8% in SC and SF conditions, respectively; *P* = 0.480).

**Fig. 8:**
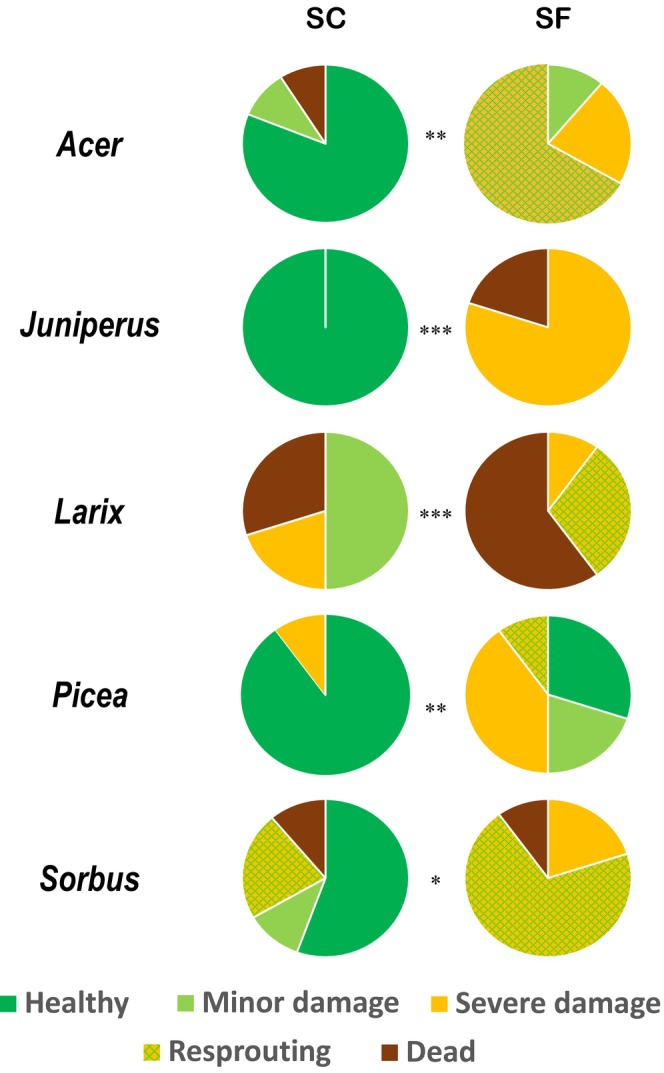
Summer mortality index (MI_summer_) per species (*Acer pseudoplatanus*, *Juniperus communis*, *Larix decidua*, *Picea abies* and *Sorbus aucuparia*) and per treatment (snow‐covered – SC‐ and snow‐free – SF‐ conditions; *n* = 10). Healthy trees (dark green) were defined as completely green; trees with minor damages were defined with some leaves to maximum one dead‐looking twig; trees with severe damages were defined as having more than one dead‐looking twig but at least one that looked alive; resprouting was defined as branches growing from the roots and death was defined when tree was completely dead (completely brown). *P*‐values, according to Kruskal–Wallis tests are indicated by an asterisk (*, ** and *** corresponding to *P* < 0.05; *P* < 0.01; *P* < 0.001, respectively).

However, a strong and significant effect of SC conditions was observed on sapling Summer Mortality Index (MI_summer_) for all species (*P* < 0.001; Fig. 8). For angiosperms, most SC trees looked healthy (e.g. 82% and 56% of trees in State 1 for *Acer* and *Sorbus*, respectively), whereas none of the SF trees were in State 1 at this time, being mainly resprouting (State 4, for 66.7% and 70% of *Acer* and *Sorbus*, respectively). Conifer saplings showed more variability across species. On the one hand, *Larix* was the most damaged species in both SC conditions (30% of dead trees) and SF conditions (60% of dead trees). On the other hand, all *Picea* and *Juniperus* saplings survived in SC conditions, exhibiting mainly State 1 with only one individual being severely damaged (State 3). Under SF conditions, all *Picea* trees survived with 90% of healthy trees in summer whereas 20% of *Juniperus* saplings died (State 5) and 80% exhibited severe damage (State 3). Overall, *Juniperus* was the most affected by snow removal, with the highest difference between snow conditions. In SF conditions, *Larix* and *Juniperus* had the highest MI_summer_.

#### Effect of spring key ecophysiological parameters on summer relative diameter growth and summer mortality index

**Fig. 9:**
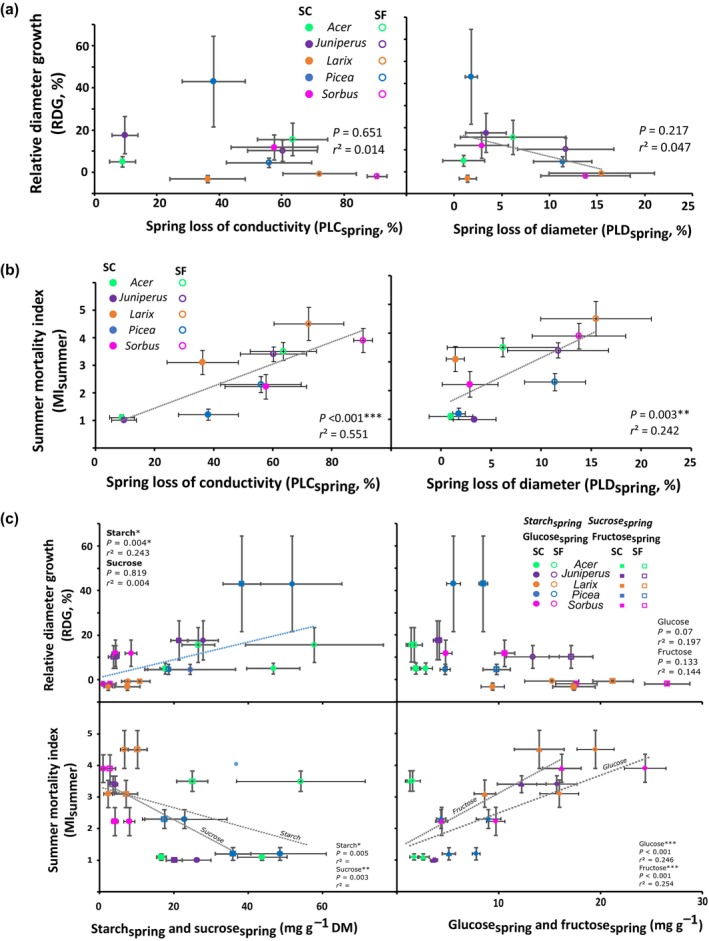
Relative diameter growth and summer mortality index depending on spring loss of conductivity and diameter as well as spring starch, sucrose, glucose and fructose contents. (a) Relative diameter growth (RDG) of snow‐covered (SC) and snow‐free (SF) saplings of the five species depending on percent spring loss of conductivity (PLC_spring_) and spring percent loss of diameter (PLD_spring_). Symbols and bars represent means and SEs, respectively. The dotted line indicates the best linear regression. (b) Summer mortality index (MI_summer_) of snow‐covered (SC) and snow‐free (SF) saplings of the five species depending on spring loss of conductivity (PLC_spring_) and spring loss of diameter (PLD_Spring_). Symbols and bars represent means and SEs, respectively. The dotted line indicates the best linear regression. (c) Relative diameter growth (RDG) and summer mortality index (MI_summer_) of snow‐covered (SC) and snow‐free (SF) saplings of the five species depending on Spring starch content (Starch_spring_), spring sucrose content (Sucrose_spring_), spring fructose content (Fructose_spring_) and spring glucose content (Glucose_spring_; in mg g^−1^). Symbols and bars represent means and SEs, respectively. The dotted line indicates the best linear regression.

Stem RDG was positively correlated with Starch_spring_ (*P* = 0.042). No significant correlation was observed between stem RDG and PLD_spring_ (*P* = 0.2172; Fig. 9a), PLC_spring_ (*P* = 0.651), nor NSCs_spring_ (*P* = 0.475) across species.

MI_summer_ was positively correlated with PLC_spring_ (*P* < 0.001), PLD_spring_ (*P* = 0.003), Glucose_spring_ (*P* < 0.001) and Fructose_spring_ (*P* < 0.001; Fig. 9b). On the opposite, MI_summer_ was negatively correlated with Sucrose_spring_ (*P* = 0.003) and Starch_spring_ (*P* = 0.012; Fig. 9c). However, no correlation with NSCs_spring_ was observed (*P* = 0.178).

**Fig. 10:**
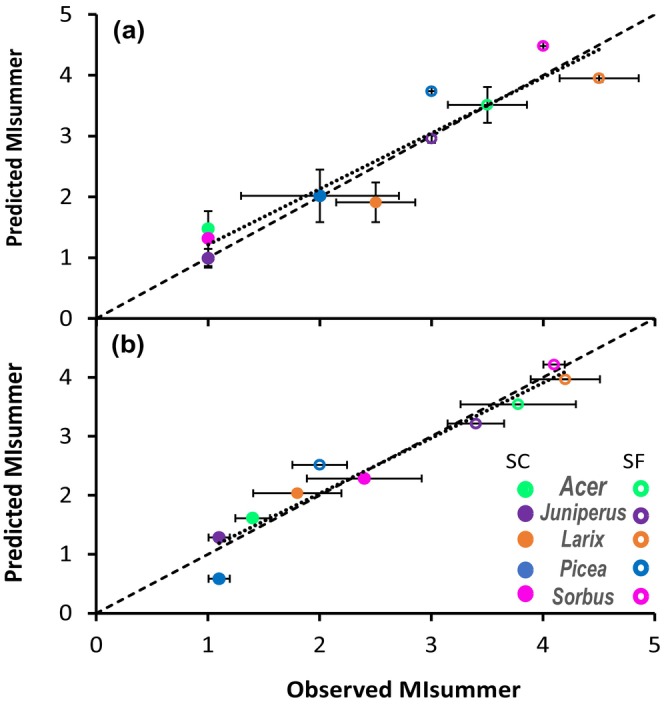
Correlation between predicted and observed values of summer mortality index (MI_summer_) according to the model based on experimental factors (snow conditions and species characteristics; a) and physiological variables (spring loss of conductivity, PLC_spring_; spring loss of diameter, PLD_spring_; spring sucrose content, Sucrose_spring_; and spring starch content, Starch_spring_; b).

Among all the variables and factors exhibiting a significant effect on MI_summer_, the best linear model predicting MI_summer_ used the following physiological variables: PLC_spring_ (*P* < 0.001), PLD_spring_ (*P* < 0.001), Sucrose_spring_ (*P* = 0.047) and Starch_spring_ (*P* = 0.005). This model was more accurate (Eff = 0.895; RMSE = 0.417; Fig. 9a) than a model simply based on experimental factors (snow conditions and species characteristics; Eff = 0.604; RMSE = 0.931). The physiological model was also relatively robust, as the RMSEP of 0.586 obtained by leave‐one‐out cross validation was close to RMSE (Fig. 10b).

#### Is there a direct link between PLD_winter_ and RDG or MI_summer_ ?

**Fig. 11:**
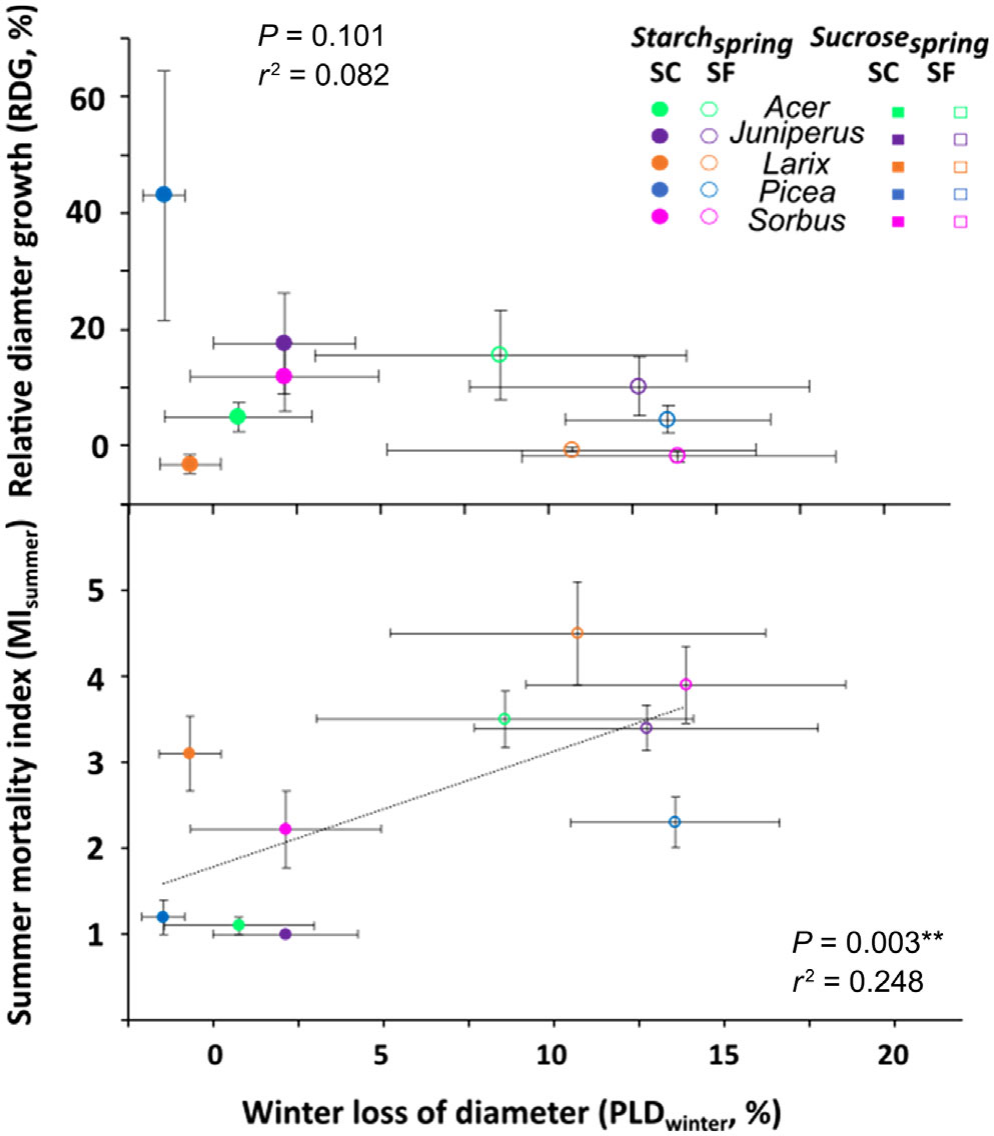
Relative stem growth (RDG) and summer mortality index (MI_summer_) of snow‐covered (SC) and snow‐free (SF) saplings of the five species depending on winter loss of diameter (PLD_winter_). Full symbols represent trees growing under control conditions and empty symbols under stressed conditions. Symbols and bars represent means and SEs, respectively. The dotted line indicates the best linear regression.

No correlation was observed between PLD_winter_ and RDG (*P* = 0.101; Fig. 11).

MI_summer_ was also correlated with PLD_winter_ (*P* = 0.003), with higher winter shrinkage corresponding to more damage. The highest PLD_winter_ observed for SF *Sorbus* (13.9%) corresponded to MI_Summer_ of 3.9 and the highest MI_summer_ was observed for *Larix* in both conditions (MI_summer_ of 3.1 and 4.5 in SC and SF conditions, respectively). No differences between angiosperm and conifer species were observed.

## Discussion

The lack of snow cover, causing a drastic change in environmental conditions during the whole winter, exposed tree and shrub saplings to higher winter drought, numerous FTE and more extreme temperatures than saplings under snow cover. We hypothesized that (1) lack of snow cover will induce frost damages on living cells and hydraulic compartments, (2) different woody species will react according to their specific physiological thresholds and (3) survival to SF winter conditions will depend on the amount of both hydraulic and cell damages and on the ability to mobilize carbohydrates to supply re‐growth.

In the light of the initial hypothesis, (1) lack of snow cover induced higher winter stress intensity (PLD_winter_) and therefore higher frost damages on living cells (PLD_spring_) or hydraulic compartments (PLC_spring_). (2) These damages were not directly correlated to the specific physiological thresholds of the different angiosperm and conifer species, neither regarding living cells nor regarding hydraulic compartments; suggesting that minimum temperature and minimum water potential were not the only factors leading to observed damages. (3) Such an approach is crucial for understanding and anticipating the regeneration and survival of young trees and shrubs in the context of climate change.

### Snow removal induced the exposure to stressful winter conditions

By removing the shelter provided by snow cover, saplings under SF conditions were exposed to more stressful environmental conditions: more freeze–thaw cycles and lower minimum temperature. Superficial soil layers (up to 10 cm depth) remained continuously frozen throughout the winter, whereas air temperature exhibited large daily fluctuations, typical conditions of winter drought (Michaelis, 1934; Pisek & Larcher, 1954; Larcher, 1972; Tranquillini, 1976, 1980).

### Maximum loss of diameter (PLD_winter_ ) as an index of winter stress intensity

Under SF conditions, trees gradually dehydrated and larger diameter shrinkage was observed in mid‐winter (PLD_winter_: 6–14%) than in SC conditions (PLD_winter_: 0–2%; Fig. 2). Snow removal, by cooling, and even freezing, soil upper layers reduced root water uptake (Charrier & Améglio, 2024), whereas removing the insulation effect of snow from dry air in the daytime (solar radiation and low air relative humidity due to the presence of snow) resulted in constant water losses (Larcher, 1963). Limited root water uptake (frozen or cold soil; Améglio *et al*., 2002; Charrier & Améglio, 2024) and high evaporative demand Charrier *et al*., 2017) will lead to a gradual reduction in water content and stem shrinkage. A shrinkage of similar magnitude (6–10%) was reported in young *Picea abies* stems exposed to freezing stress (Zweifel & Häsler, 2000). Mid‐winter stem shrinkage is due to the combined effect of two factors: winter drought and freeze‐induced dehydration (Zweifel & Häsler, 2000; Charrier *et al*., 2017).

Winter drought, under the control of warm daytime temperature and low vapor pressure deficit, induced the dehydration of stem tissues, especially bark living cells (Zweifel & Häsler, 2000; Charrier *et al*., 2017). As living cells lose their turgor, a progressive stem shrinkage is observed (Améglio & Cruiziat, 1992; Zweifel & Häsler, 2000; Zweifel *et al*., 2006; Lamacque *et al*., 2020). Furthermore, extracellular ice formation induced stem shrinkage by freeze‐induced dehydration due to the relation between water potential of ice and temperature (Hansen & Beck, 1988; Améglio *et al*., 2001; Oogathoo *et al*., 2024). The shrinkage of living cells was, however, reversible (Sakai & Larcher, 1987; Améglio *et al*., 2001; Charra‐Vaskou *et al*., 2016; Lintunen *et al*., 2017). When stress is released (positive temperature, well‐hydrated soil and moderate evapotranspiration demand), liquid water flows along the osmotic gradient, back to the cell symplast, allowing the bark to recover its initial size when all cells are intact (Améglio *et al*., 2001).

### Snow removal induced hydraulic damages (PLC_spring_ )

Winter drought induced significant PLC_spring_ by disturbing water balance between root absorption and evapotranspiration, resulting in low sap water potential and the creation of embolism (Tyree & Cochard, 1996; Mayr *et al*., 2002). Loss of hydraulic conductivity can be further aggravated by exposure to an increased number of FTE (Sperry & Sullivan, 1992; Sperry *et al*., 1994; Mayr *et al*., 2003, 2007; Ball *et al*., 2006; Charrier *et al*., 2015; Charra‐Vaskou *et al*., 2016) and to lower minimum temperature (Pittermann & Sperry, 2003; Charra‐Vaskou *et al*., 2023), particularly in SF conditions (Fig. 6a). Mountain environments are typical locations where the interaction between frost nights and warmer days leads to the generation of cavitation and embolism events (Charrier *et al*., 2017, 2021).

Interestingly, although vessel‐bearing species (i.e. angiosperms) are usually more prone to FT induced embolism than tracheid‐bearing species (i.e. conifers; Davis *et al*., 1999; Pittermann & Sperry, 2003; Mayr & Sperry, 2010; Charrier *et al*., 2013a, 2013b), we did not observe this relationship in our experiment (Fig. 3). The sampling date in spring was probably after some species refilled their xylem from embolism. *Acer* was indeed generating positive pressure, as sap exudation was observed after cutting stems for sampling (Kramer, 1940; O'Malley, 1979; O'Malley & Milburn, 1983; F. W. Ewers *et al*., 2001; B. E. Ewers *et al*., 2001). Xylem positive pressure can be generated by root pressure through osmotic pressurization of the symplasmic compartments of root cells that propagates toward the upper parts of the plant (Fisher *et al*., 1997) or by stem pressure which involves water and carbohydrate fluxes between bark, xylem parenchyma cells and vessel lumens, in response to freeze–thaw cycles (Bozonnet *et al*., 2022, 2024). *Picea* is also known to remobilize external water to restore hydraulic conductivity but, in SF conditions, the water from melting snow could not be involved (Mayr *et al*., 2014).

### Snow removal induced cellular damages (PLD_spring_ )

In spring, when stem diameter cannot recover its autumn value despite favorable conditions, damages to living cells are expected (Améglio *et al*., 2001; Lintunen *et al*., 2015, 2017). Indeed, PLD_spring_ is considered an index of living cell mortality (Lintunen *et al.*, 2015; Lamacque *et al*., 2020; Andriantelomanana *et al*., 2024) and was indeed significantly correlated to the reference electrolyte leakage method (Fig. 4).

PLD_spring_ was higher in SF conditions for all species, suggesting that stress exposure caused substantial cellular damage (Fig. 3). However, the maximum frost hardiness of any species was reached in any of the species this winter (minimum temperature −21°C vs frost hardiness between −29°C for *Larix* and −80°C for *Juniperus*; Larter *et al*., this special issue). Although a small proportion of cells may have been damaged, the minimum temperature was not the main factor leading to the observed damage. However, rapid temperature changes during freezing and thawing may have caused substantial damage. Daily temperature amplitudes can reach up to 30°C at the same site (Mayr *et al*., 2006b), with maximum rates reaching 15 K h^−1^ for cooling and thawing (Charrier *et al*., 2017). These rates were much slower than the rates inducing intracellular freezing (−48 K h^−1^; Levitt & Levitt, 1958; Siminovitch *et al*., 1978) or water influx during thawing (+20 K min^−1^; Sakai *et al*., 1968), respectively, indicating that temperature variation was not the main factor leading to cell damage.

On the one hand, dehydration stress can have further exacerbated cell damage. Beyond a certain dehydration threshold, the cells cannot withstand osmotic stress and lose their integrity, resulting in an irreversible loss of diameter (Lamacque *et al*., 2020, 2022). These complex dynamics and interactions between factors are usually underestimated when assessing damage to living cells in harsh ecosystems. On the other hand, cell damage can have exacerbated hydraulic damage through frost damage on the root system. Frost hardiness in cold‐acclimated roots is usually measured below −8°C, even in relatively cold‐sensitive species such as *Olea europaea, Quercus ilex* and *Juglans regia* (Sakai & Larcher, 1987; Charrier *et al*., 2013a, 2013b; Ambroise *et al*., 2020), or below −16°C in *Picea glauca* (Carles *et al*., 2012). In our study, the minimum temperature at a depth of 20 cm only reached −5°C for short periods, suggesting that frost‐induced damage to the root systems was not substantial. A simulated thaw followed by prolonged freezing induced a hydraulic failure and decline in *Betula alleghaniensis*, but the plants were exposed to −10°C for 10 wk (Cox & Zhu, 2003). In our study, most of the SF trees were able to rehydrate and regrow, even though hydraulic damage and freezing damage to the roots could both have contributed to delayed and reduced growth.

### Snow removal altered carbon metabolism

Overall, in SF conditions, no significant differences were observed in NSCs content but higher Glucose_spring_ and Fructose_spring_ and lower Sucrose_spring_ and Starch_spring_ than in SC conditions were observed (Fig. 5). The inter‐conversion of starch into soluble carbohydrates is a typical pattern associated with cold acclimation and deacclimation (Sakai, 1966; Améglio *et al*., 2004; Baffoin *et al*., 2021). Low starch and high hexose (glucose and fructose) would suggest that the saplings had delayed cold deacclimation dynamics, that is, were still in an acclimated state. The lower minimum temperature, the main driver of cold deacclimation, under SF conditions could indeed have delayed starch resynthesis in late winter (Charrier *et al*., 2018). Such a delay could also explain the phenological delay observed in bud burst (data not shown but see Frey, 1983; Wipf & Rixen, 2010; Shakhmatov *et al*., 2022). Interestingly, species most affected by winter conditions (*Sorbus* and *Larix* in both conditions, *Juniperus* and *Picea* in SF conditions) showed high Glucose_spring_ and Fructose_spring_. However, the dynamics of sucrose is usually closely linked and connected to that of hexose (Deslauriers *et al*., 2021). Sucrose is the main mobile form of soluble carbohydrate (Bonhomme *et al*., 2010; Dominguez & Nittlya, 2022), and its redistribution to supply growth can be altered by xylem embolism, therefore reducing growth potential (Bonhomme *et al*., 2010).

### Snow removal determined growth rate (RDG)

RDG was lower in SF conditions than in SC for all species, as observed by Domisch *et al*. (2018, 2019). Interestingly, RDG was negatively correlated with PLD_spring_ and positively correlated with Starch_spring_ but not with PLC_spring_ (Fig. 9a). This result suggests that although cellular damage affected growth dynamics, hydraulic failure was not the main factor reducing the magnitude of growth. Growth was indeed more closely correlated with the mortality of living cells, since their active division and expansion are the foundation for growth (Meyer & Boyer, 1972). Among the plant processes affected by mild water stress, cell growth is probably the most sensitive, as turgor pressure is considered to play a critical role in cell growth (Lockhart, 1965; Hsiao *et al*., 1976). The saplings with higher growth also had a higher Starch_spring_ which could supply growth processes (Sulpice *et al*., 2009). Overall, the correlations between RDG and the different ecophysiological parameters were weak, suggesting that many other factors are likely to be involved in determining the magnitude of growth (Rauscher *et al*., 1990). Furthermore, a better spatiotemporal resolution of NCS dynamics, such as the remobilization of reserves from the root system, would better reflect growth potential (Simard *et al*., 2013).

### Snow removal induced mortality (MI_summer_ )

For all species, MI_summer_ was higher in SF than in SC conditions (Fig. 10) as also observed by Drescher & Thomas (2013). MI_summer_ was positively correlated with PLC_spring_, PLD_spring_, Glucose_spring_ and Fructose_spring_ and negatively with Starch_spring_ and Sucrose_spring_ (Fig. 9b,c). Finally, PLC_spring_, PLD_spring_, Sucrose_spring_ and Starch_spring_ allow predicting the vitality of saplings in the subsequent vegetation period.

PLC_spring_ reflects how the hydraulic compartment can supply distal organs at the onset of growth (Peguero‐Pina *et al*., 2011), thus determining sapling decline later in the growing season (Fig. 9b). Drought‐induced embolism is a key parameter for predicting mortality with physiological thresholds observed at 88% PLC for angiosperms (Barigah *et al*., 2013; Urli, 2013; Adams *et al*., 2017) and 50% PLC for conifers (Brodribb & Cochard, 2009; Adams *et al*., 2017; Choat *et al*., 2018). However, under SF conditions, only *Acer* remained below this threshold (Fig. 9b). Higher PLC than these thresholds are usually observed during winter stress without implying tree death (Mayr *et al*., 2003, 2014; Mayr & Charra‐Vaskou, 2007). Indeed, higher PLC is not as critical during winter for deciduous plants because they do not transpire, and even for evergreens, evaporative demand is usually lower than during the growing season; transpiration rate can be controlled by stomatal closure, and no water is needed for growth processes. In any case, PLC_spring_ is a good predictive indicator of upcoming summer mortality. Despite PLC higher than 50%, most conifers survived, except *Larix*. For angiosperms, PLC was either below (*Acer*) or above (*Sorbus*) the 88% threshold, but the severely damaged or even dead saplings were able to regrow from the root system (resprouting).

PLD_spring_, corresponding to living cell mortality, was a key physiological indicator determining MI_summer_ and RDG (Fig. 9b). PLD was considered to represent the emptying of bark elastic reservoir during drought stress (Lamacque *et al*., 2020), although both xylem and bark elastic tissues could contribute to freeze‐induced shrinkage (Améglio *et al*., 2001). Dehydration of living cells may lead to cell mortality by mechanical damage, collapse or biochemical damage (Andriantelomanana *et al*., 2024) and to tree mortality as they contribute to growth via the cambium and to carbon allocation via the phloem (Lanner, 2002). A significant damage to bark living cells deprives the tree of a large water reservoir (Andriantelomanana *et al*., 2024). Lethal PLD was highly variable across species: 7.58% for *Populus* (Andriantelomanana *et al*., 2024) or 21.3% for *Lavender* (Lamacque *et al*., 2020). In our interspecific studies, the PLD_spring_ that significantly increased mortality risk was in agreement, comprised between 5% and 10% (Fig. 9b).

The relation between MI_summer_ and nonstructural carbohydrate was complex (Fig. 8c). Although the total amount of NSCs did not appear critical, the ratio between osmotically active compounds in the cell (glucose and fructose) and osmotically neutral (starch) or mobile form (fructose) seems critical (*P* = 0.001). The low Starch_spring_ could be a result of a depletion of reserves due to winter drought and potentially contribute to reduced vitality or increased tree morbidity (McDowell *et al*., 2008; Earles *et al*., 2018). However, total NSCs were not significantly affected by snow removal suggesting that the relation with mortality is not limited by source limitation but rather by sink strength, as meristem cells were damaged or the ability to translocate sugars to the sink cells through hydraulic limitation. Sink limitation is indeed a key factor explaining plant survival at treeline (Hoch *et al*., 2002; Dolezal *et al*., 2019). However, how the temporal pattern of NSCs dynamics and remobilization of belowground reserves are affected should be investigated in SF conditions to better understand the consequences for sapling survival.

### Predicting MI_summer_ based on spring ecophysiological parameters

Summer sapling mortality can be simply explained by the interaction between experimental factors (*P* < 0.001) and species (*P* < 0.01). However, to gain insights into the mechanism leading to death, we assessed the potential of physiological variables to predict MI_summer_ with better accuracy than experimental factors. The best model by integrating the additive effect of PLD_spring_. PLC_spring_, Sucrose_spring_ and Starch_spring_ showed that tree mortality is likely to result from a combined effect of hydraulic failure, living cell mortality and perturbation in carbon allocation (Sala *et al*., 2010; McDowell, 2011; Lamacque *et al*., 2022). Furthermore, although living cell mortality and hydraulic failure can affect starch and sucrose dynamics, starch and sucrose had a significant effect, showing that NSCs dynamics is a relevant factor in explaining mortality.

### Interspecific differences in resistance and recovery

Overall, across the five species, a significant correlation was observed between PLC_spring_ and PLD_spring_ (Fig. 6c), indicating that species are able to combine stress resistance for both compartments (*P* = 0.030, Fig. 6c; also observed by Lintunen *et al*., 2013). Contrasting effects of snow removal were observed across angiosperm species. *Sorbus* was severely damaged, whereas *Acer* remained much less affected, although *Acer* was much closer to its altitudinal limit. Across conifers, *Larix* was the less resistant species in SF conditions, whereas the drought and frost‐resistant *Juniperus* (P_50_ = −6.57 MPa, LT_50_ = −80°C; Larter *et al*., this special issue) showed relatively low PLC_spring_ in both conditions and moderate living cell mortality in SF conditions (Fig. 6a). *Picea* showed a globally high resistance against SF conditions with the lowest PLC_spring_ and moderate PLD_spring_. While the overall conditions at our study site were comparable to those of natural habitats, the saplings may have been exposed to harsher climatic hazards as the site was open with no tall trees to provide shelter.

Sensitive species exhibited other strategies to recover from stress and survive, thus showing a certain form of resilience (Lloret *et al*., 2011). Under SF conditions *Sorbus* showed no growth with high PLC_spring_, PLD_spring_, Glucose_spring_ and Starch_spring_ (Fig. 9a,c) and high mortality (Fig. 9b). However, early and vigorous resprouting from the root system was systematically observed. This strategy does not affect the survival of *Sorbus* in the long term, although it prevents the individual from reaching tree size (Myking *et al*., 2013; Beloiu *et al*., 2020). Thus, *Sorbus* trees are likely to exhibit low stature under the absence of snow cover. However, the cumulative effect of successive SF winters could jeopardize this species by depleting carbon reserves in roots, although sprouting species have a higher root : shoot ratio (Sakai *et al*., 1997). *Acer* SF saplings showed severe damage to aboveground organs with a late resprouting. Surprisingly RDG was low in SC conditions despite very low PLD_spring_ and PLC_spring_ (Fig. 9b). Contrarily to *Sorbus*, *Acer* exhibits an active recovery mechanism to restore physiological functioning at the cost of limiting growth.

*Larix* was the most affected in both SF and SC conditions with high MI_summer_ and very low growth. This species exhibits large stress legacy effects by reducing growth (Vitasse *et al*., 2019; Du *et al*. 2024). No recovery strategy was observed in *Larix*, suggesting that the regeneration of this species in reduced snow conditions is the highest across the studied species. On the contrary, *Juniperus* was completely healthy in summer in SC conditions (MI_summer_ 1.0; Figs 8, 9b) but showed severe damage in SF conditions (in most cases only one stem remaining alive). Finally, *Juniperus* shrubs were relatively resistant with a high growth rate, but once damaged could not recover. Under winter stress, *Juniperus*, by maintaining radial growth and limiting primary growth, thus exhibits a conservative strategy (Camarero *et al*., 2024). *Picea* was the most affected in growth by snow removal, exhibiting the highest growth in SC conditions (18.3% with relatively low PLD_spring_) and the second lowest growth in SF conditions (−0.5%, with relatively high PLD_spring_; Fig. 8a). Although highly resistant to snow removal, *Picea* strongly limited growth after stress (Lamhamedi *et al*., 2001). *Picea* is able to recover PLC_spring_ by refilling process at the cost of drought (Mayr *et al*., 2002, 2003, 2014).

In our study, RDG and MI_summer_ of the five species were only partially correlated to growth form and functional types. The shrub species *Juniperus* did not really differ in the physiological parameters from the tall trees but showed the highest MI_summer_ difference between treatments (Fig. 8) with a low MI_summer_ in SC conditions and a high MI_summer_ in SF conditions (particularly taking into account that no resprouting strategy occurred). This result is in accordance with the observation that most Alpine shrubs depend on the protective snow cover. Nevertheless, tall trees (particularly deciduous tall trees) showed also a high MI_summer_ in SF conditions, probably as in our study saplings, more winter drought sensitive than mature trees (Mayr & Charra‐Vaskou, 2007), were used.

Interestingly, the highest PLC_spring_ was reported for the 3 deciduous species (*Sorbus, Larix* and *Acer*), while contrasting results were observed regarding PLD_spring_ and overall high MI_summer_ was observed for deciduous species. Thus, despite these species shedding their leaves in autumn, freezing temperatures, FTEs and high winter drought cause complex stress patterns leading to embolism and living cell damage. Contrasting results were observed for the two analyzed conifer species with narrow tracheids. While *Picea* showed the lowest MI_summer_, *Larix*, as a drought‐sensitive species, showed the highest. In our study, drought resistance mechanisms such as narrow conduits, leaf fall in autumn or a shrubby form seem to have only a weak influence on sapling resistance in SF conditions, and other mechanisms, such as refilling (*Picea*), positive stem pressure (*Acer*) or resprouting (*Sorbus*, *Acer*), may have a major impact.

To conclude, in the context of reduced snow cover, saplings are at risk of increased mortality and reduced growth (Frey, 1983; Drescher & Thomas, 2013). Using noninvasive sensors combined with destructive sampling in a large range of growth form and functional types (shrubs and trees, evergreen and deciduous, angiosperm and deciduous), we identified the key ecophysiological parameters involved. Mortality was predicted based on physiological parameters suggesting that interspecific differences arise from contrasting resistance and recovery mechanisms that maintain hydraulic integrity, sustain cell vitality and enable carbohydrate translocation for growth. Nevertheless, the interplay between these processes is complex and further studies on their spatial and temporal dynamics are needed. In the context of climate change, with the predicted reduction of snow cover in height and duration, the survival of young trees and shrubs exposed to successive SF winters is likely to endanger regeneration in mountain vegetation.

## Competing interests

None declared.

## Author contributions

KCV, GC, TA and SM built up the experimental design. KCV, GC and AG set up the experiment, monitored it and collected the samples. KCV, GC and TA analyzed the data and built the statistical model. KCV wrote the manuscript, with help of GC and SM and inputs from all authors.

## Disclaimer

The New Phytologist Foundation remains neutral with regard to jurisdictional claims in maps and in any institutional affiliations.

## Supporting information


**Dataset S1** Synthetic dataset of all values used in the manuscript for each individual.

**Fig. S1** Stem diameter variations of one representative individual per species and per treatment (snow‐covered and snow‐free conditions) as well as mean stem and soil temperature and soil water potential during 12 months.
**Fig. S2** Correlation between visualized cellular mortality and spring loss of diameter of snow‐covered and snow‐free saplings of the five species.
Please note: Wiley is not responsible for the content or functionality of any Supporting Information supplied by the authors. Any queries (other than missing material) should be directed to the *New Phytologist* Central Office.

## Data Availability

The synthetic data are available in the Supporting Information Dataset (S1).

## References

[nph70926-bib-0001] Adams HD , Zeppel MJ , Anderegg WR , Hartmann H , Landhäusser SM , Tissue DT , Zeppel MJB , Anderegg WRL , Huxman TE , Hudson PJ *et al*. 2017. A multi‐species synthesis of physiological mechanisms in drought‐induced tree mortality. Nature Ecology & Evolution 1: 1285–1291. 29046541 10.1038/s41559-017-0248-x

[nph70926-bib-0002] Akaike H . 1974. A new look at the statistical model identification. IEEE Transactions on Automatic Control 19: 716–723.

[nph70926-bib-0003] Ambroise V , Legay S , Guerriero G , Hausman JF , Cuypers A , Sergeant K . 2020. The roots of plant frost hardiness and tolerance. Plant and Cell Physiology 61: 3–20. 31626277 10.1093/pcp/pcz196 PMC6977023

[nph70926-bib-0004] Améglio T , Bodet C , Lacointe A , Cochard H . 2002. Winter embolism, mechanisms of xylem hydraulic conductivity recovery and springtime growth patterns in walnut and peach trees. Tree Physiology 22: 1211–1220. 12464574 10.1093/treephys/22.17.1211

[nph70926-bib-0005] Améglio T , Cochard H , Ewers FW . 2001. Stem diameter variations and cold hardiness in walnut trees. Journal of Experimental Botany 52: 2135–2142. 11604452 10.1093/jexbot/52.364.2135

[nph70926-bib-0006] Améglio T , Cruiziat P . 1992. Alternance tension‐pression de la seve dans le xyleme chez le Noyer pendant l'hiver: role des temperatures. Comptes Rendus De L'Académie des Sciences. Série III, Sciences De La Vie 315: 429–435.

[nph70926-bib-0007] Améglio T , Decourteix M , Alves G , Valentin V , Sakr S , Julien JL , Petel G , Guilliot A , Lacointe A . 2004. Temperature effects on xylem sap osmolarity in walnut trees: evidence for a vitalistic model of winter embolism repair. Tree Physiology 24: 785–793. 15123450 10.1093/treephys/24.7.785

[nph70926-bib-0008] Andriantelomanana T , Améglio T , Delzon S , Cochard H , Herbette S . 2024. Unpacking the point of no return under drought in poplar: insight from stem diameter variation. New Phytologist 242: 466–478. 38406847 10.1111/nph.19615

[nph70926-bib-0009] Arora R . 2018. Mechanism of freeze‐thaw injury and recovery: A cool retrospective and warming up to new ideas. Plant Science 270: 301–313. 29576084 10.1016/j.plantsci.2018.03.002

[nph70926-bib-0010] Baffoin R , Charrier G , Bouchardon AE , Bonhomme M , Améglio T , Lacointe A . 2021. Seasonal changes in carbohydrates and water content predict dynamics of frost hardiness in various temperate tree species. Tree Physiology 41: 1583–1600. 33611596 10.1093/treephys/tpab033

[nph70926-bib-0011] Ball MC , Canny MJ , Huang CX , Egerton JJG , Wolfe J . 2006. Freeze/thaw‐induced embolism depends on nadir temperature: the heterogeneous hydration hypothesis. Plant, Cell & Environment 29: 729–745. 10.1111/j.1365-3040.2005.01426.x 17087458

[nph70926-bib-0012] Bannister P , Maegli T , Dickinson KJ , Halloy SR , Knight A , Lord JM , Mark AF , Spencer KL . 2005. Will loss of snow cover during climatic warming expose New Zealand alpine plants to increased frost damage? Oecologia 144: 245–256. 15891822 10.1007/s00442-005-0087-3

[nph70926-bib-0155] Baranger A , Cordonnier T , Charrier G , Delzon S , Larter M , Martin‐StPaul NK , Kunstler G . In Press. Living on the edge–physiological tolerance to frost and drought explains range limits of 35 European tree species. Ecography: e07528. doi: 10.1111/ecog.07528

[nph70926-bib-0014] Barigah TS , Charrier O , Douris M , Bonhomme M , Herbette S , Améglio T , Fichot R , Brignolas F , Cochard H . 2013. Water stress‐induced xylem hydraulic failure is a causal factor of tree mortality in beech and poplar. Annals of Botany 112: 1431–1437. 24081280 10.1093/aob/mct204 PMC3806533

[nph70926-bib-0015] Beloiu M , Stahlmann R , Beierkuhnlein C . 2020. High recovery of saplings after severe drought in temperate deciduous forests. Forests 11: 546.

[nph70926-bib-0150] Bonhomme M , Peuch M , Ameglio T , Rageau R , Guilliot A , Decourteix M , Alves G , Sakr S , Lacointe A . 2010. Carbohydrate uptake from xylem vessels and its distribution among stem tissues and buds in walnut (*Juglans regia* L.). Tree Physiology 30: 89–102. 19955192 10.1093/treephys/tpp103

[nph70926-bib-0016] Bozonnet C , Saudreau M , Badel E , Améglio T , Charrier G . 2022. Multi‐physics modelling of freeze‐thaw cycles effects on tree branches. In 10th Plant Biomechanics Conference.

[nph70926-bib-0017] Bozonnet C , Saudreau M , Badel E , Charrier G , Améglio T . 2024. On the mechanism for winter stem pressure build‐up in walnut trees. Tree Physiology 44: tpae037. 38531772 10.1093/treephys/tpae037

[nph70926-bib-0018] Briceño VF , Harris‐Pascal D , Nicotra AB , Williams E , Ball MC . 2014. Variation in snow cover drives differences in frost resistance in seedlings of the alpine herb *Aciphylla glacialis* . Environmental and Experimental Botany 106: 174–181.

[nph70926-bib-0019] Brodribb TJ , Cochard H . 2009. Hydraulic failure defines the recovery and point of death in water‐stressed conifers. Plant Physiology 149: 575–584. 19011001 10.1104/pp.108.129783 PMC2613726

[nph70926-bib-0020] Burghardt M , Riederer M . 2003. Ecophysiological relevance of cuticular transpiration of deciduous and evergreen plants in relation to stomatal closure and leaf water potential. Journal of Experimental Botany 54: 1941–1949. 12815029 10.1093/jxb/erg195

[nph70926-bib-0021] Cai SY , Li QD . 2024. Snow cover dynamics: impacts on soil moisture and plant growth in temperate ecosystems. Molecular Soil Biology 15: 109–117.

[nph70926-bib-0022] Camarero JJ , Pizarro M , Gernandt DS , Gazol A . 2024. Smaller conifers are more resilient to drought. Agricultural and Forest Meteorology 350: 109993.

[nph70926-bib-0149] Carles S , Lamhamedi MS , Beaulieu J , Stowe DC , Margolis HA . 2012. Genetic parameters of morphological and physiological characteristics of containerized white spruce (*Picea glauca* [Moench.] Voss) seedlings. Tree Genetics & Genomes 8: 39–51.

[nph70926-bib-0023] Charra‐Vaskou K , Badel E , Burlett R , Cochard H , Delzon S , Mayr S . 2012. Hydraulic efficiency and safety of vascular and non‐vascular components in Pinus pinaster leaves. Tree Physiology 32: 1161–1170. 22907978 10.1093/treephys/tps071

[nph70926-bib-0024] Charra‐Vaskou K , Badel E , Charrier G , Ponomarenko A , Bonhomme M , Foucat L , Améglio T . 2016. Cavitation and water fluxes driven by ice water potential in *Juglans regi*a during freeze–thaw cycles. Journal of Experimental Botany 67: 739–750. 26585223 10.1093/jxb/erv486 PMC4737071

[nph70926-bib-0025] Charra‐Vaskou K , Lintunen A , Améglio T , Badel E , Cochard H , Mayr S , Charrier G . 2023. Xylem embolism and bubble formation during freezing suggest complex dynamics of pressure in *Betula pendula* stems. Journal of Experimental Botany 74: 5840–5853. 37463327 10.1093/jxb/erad275

[nph70926-bib-0026] Charrier G , Améglio T . 2024. Dynamic modeling of stem water content during the dormant period in walnut trees. Tree Physiology 44: tpad128. 37847599 10.1093/treephys/tpad128

[nph70926-bib-0027] Charrier G , Charra‐Vaskou K , Kasuga J , Cochard H , Mayr S , Améglio T . 2014. Freeze‐thaw stress: effects of temperature on hydraulic conductivity and ultrasonic activity in ten woody angiosperms. Plant Physiology 164: 992–998. 24344170 10.1104/pp.113.228403 PMC3912121

[nph70926-bib-0028] Charrier G , Cochard H , Améglio T . 2013a. Evaluation of the impact of frost resistances on potential altitudinal limit of trees. Tree Physiology 33: 891–902. 24052567 10.1093/treephys/tpt062

[nph70926-bib-0029] Charrier G , Lacointe A , Améglio T . 2018. Dynamic modeling of carbon metabolism during the dormant period accurately predicts the changes in frost hardiness in walnut trees *Juglans regia* L. Frontiers in Plant Science 9: 1746. 30568664 10.3389/fpls.2018.01746 PMC6290248

[nph70926-bib-0030] Charrier G , Martin‐StPaul N , Damesin C , Delpierre N , Hänninen H , Torres‐Ruiz JM , Davi H . 2021. Interaction of drought and frost in tree ecophysiology: rethinking the timing of risks. Annals of Forest Science 78: 1–15.

[nph70926-bib-0031] Charrier G , Nolf M , Leitinger G , Charra‐Vaskou K , Losso A , Tappeiner U , Améglio T , Mayr S . 2017. Monitoring of freezing dynamics in trees: a simple phase shift causes complexity. Plant Physiology 173: 2196–2207. 28242655 10.1104/pp.16.01815 PMC5373037

[nph70926-bib-0032] Charrier G , Poirier M , Bonhomme M , Lacointe A , Améglio T . 2013b. Frost hardiness in walnut trees (*Juglans regia* L.): how to link physiology and modelling? Tree Physiology 33: 1229–1241. 24271086 10.1093/treephys/tpt090

[nph70926-bib-0033] Charrier G , Pramsohler M , Charra‐Vaskou K , Saudreau M , Améglio T , Neuner G , Mayr S . 2015. Ultrasonic emissions during ice nucleation and propagation in plant xylem. New Phytologist 207: 570–578. 25756189 10.1111/nph.13361 PMC5024006

[nph70926-bib-0034] Choat B , Brodribb TJ , Brodersen CR , Duursma RA , López R , Medlyn BE . 2018. Triggers of tree mortality under drought. Nature 558: 531–539. 29950621 10.1038/s41586-018-0240-x

[nph70926-bib-0035] Comerford DP , Schaberg PG , Templer PH , Socci AM , Campbell JL , Wallin KF . 2013. Influence of experimental snow removal on root and canopy physiology of sugar maple trees in a northern hardwood forest. Oecologia 171: 261–269. 22752211 10.1007/s00442-012-2393-x

[nph70926-bib-0036] Cox RM , Zhu XB . 2003. Effects of simulated thaw on xylem cavitation, residual embolism, spring dieback and shoot growth in yellow birch. Tree Physiology 23: 615–624. 12750054 10.1093/treephys/23.9.615

[nph70926-bib-0037] Cruiziat P , Cochard H , Améglio T . 2002. Hydraulic architecture of trees: main concepts and results. Annals of Forest Science 59: 723–752.

[nph70926-bib-0038] Daubenmire RF . 1945. An improved type of precision dendrometer.

[nph70926-bib-0039] Davis SD , Sperry JS , Hacke UG . 1999. The relationship between xylem conduit diameter and cavitation caused by freezing. American Journal of Botany 86: 1367–1372. 10523278

[nph70926-bib-0040] De Swaef T , De Schepper V , Vandegehuchte MW , Steppe K . 2015. Stem diameter variations as a versatile research tool in ecophysiology. Tree Physiology 35: 1047–1061. 26377875 10.1093/treephys/tpv080

[nph70926-bib-0041] Deslauriers A , Garcia L , Charrier G , Buttò V , Pichette A , Paré M . 2021. Cold acclimation and deacclimation in wild blueberry: direct and indirect influence of environmental factors and non‐structural carbohydrates. Agricultural and Forest Meteorology 301: 108349.

[nph70926-bib-0042] Deslauriers A , Rossi S , Anfodillo T . 2007. Dendrometer and intra‐annual tree growth: what kind of information can be inferred? Dendrochronologia 25: 113–124.

[nph70926-bib-0043] DeSoto L , Cailleret M , Sterck F , Jansen S , Kramer K , Robert EM , Martínez‐Vilalta J . 2020. Low growth resilience to drought is related to future mortality risk in trees. Nature Communications 11: 545. 10.1038/s41467-020-14300-5 PMC6987235 31992718

[nph70926-bib-0044] Dobbert S , Pape R , Löffler J . 2022. The application of dendrometers to alpine dwarf shrubs–a case study to investigate stem growth responses to environmental conditions. Biogeosciences 19: 1933–1958.

[nph70926-bib-0045] Dolezal J , Kopecky M , Dvorsky M , Macek M , Rehakova K , Capkova K , Altman J . 2019. Sink limitation of plant growth determines tree line in the arid Himalayas. Functional Ecology 33: 553–565.

[nph70926-bib-0153] Dominguez PG , Niittylä T . 2022. Mobile forms of carbon in trees: metabolism and transport. Tree Physiology 42: 458–487. 34542151 10.1093/treephys/tpab123 PMC8919412

[nph70926-bib-0046] Domisch T , Martz F , Repo T , Rautio P . 2018. Winter survival of Scots pine seedlings under different snow conditions. Tree Physiology 38: 602–616. 29040799 10.1093/treephys/tpx111

[nph70926-bib-0047] Domisch T , Martz F , Repo T , Rautio P . 2019. Let it snow! Winter conditions affect growth of birch seedlings during the following growing season. Tree Physiology 39: 544–555. 30517759 10.1093/treephys/tpy128

[nph70926-bib-0048] Drescher M , Thomas SC . 2013. Snow cover manipulations alter survival of early life stages of cold‐temperate tree species. Oikos 122: 541–554.

[nph70926-bib-0049] Du M , Xu C , Wang A , Lv P , Xu Z , Zhang X . 2024. Different drought recovery strategy between Larix spp. and *Quercus mongolica* in temperate forests. Science of the Total Environment 938: 173521. 38802012 10.1016/j.scitotenv.2024.173521

[nph70926-bib-0050] Earles JM , Knipfer T , Tixier A , Orozco J , Reyes C , Zwieniecki MA , McElrone AJ . 2018. *In vivo* quantification of plant starch reserves at micrometer resolution using X‐ray micro CT imaging and machine learning. New Phytologist 218: 1260–1269. 10.1111/nph.15068 29516508

[nph70926-bib-0052] Ewers BE , Oren R , Phillips N , Strömgren M , Linder S . 2001. Mean canopy stomatal conductance responses to water and nutrient availabilities in *Picea abies* and *Pinus taeda* . Tree Physiology 21: 841–850. 11498331 10.1093/treephys/21.12-13.841

[nph70926-bib-0053] Ewers FW , Ameglio T , Cochard H , Beaujard F , Martignac M , Vandame M , Bodet C , Cruiziat P . 2001. Seasonal variation in xylem pressure of walnut trees: root and stem pressures. Tree Physiology 21: 1123–1132. 11581019 10.1093/treephys/21.15.1123

[nph70926-bib-0054] Fisher JB , Guillermo Angeles A , Ewers FW , Lopez‐Portillo J . 1997. Survey of root pressure in tropical vines and woody species. International Journal of Plant Sciences 158: 44–50.

[nph70926-bib-0055] Francon L , Ameglio T , Roussel E , Bottraud IT , Charrier G , Corona C . 2018. Wood formation in rhododendrons at the stress line: take a shrub to the limit. In: Colloque Wood formation and tree adaptation to climate, Le stadium. Orléans, France: FRA, pp. A0. Ha‐l‐01844912.

[nph70926-bib-0056] Frey W . 1983. The influence of snow on growth and survival of planted trees. Arctic and Alpine Research 15: 241–251.

[nph70926-bib-0058] Ganthaler A , Mayr S . 2021. Subalpine dwarf shrubs differ in vulnerability to xylem cavitation: an innovative staining approach enables new insights. Physiologia Plantarum 172: 2011–2021. 33866574 10.1111/ppl.13429

[nph70926-bib-0059] Gerdol R , Siffi C , Iacumin P , Gualmini M , Tomaselli M . 2013. Advanced snowmelt affects vegetative growth and sexual reproduction of *Vaccinium myrtillus* in a sub‐alpine heath. Journal of Vegetation Science 24: 569–579.

[nph70926-bib-0060] Groffman PM , Driscoll CT , Fahey TJ , Hardy JP , Fitzhugh RD , Tierney GL . 2001. Colder soils in a warmer world: a snow manipulation study in a northern hardwood forest ecosystem. Biogeochemistry 56: 135–150.

[nph70926-bib-0061] Hacke UG , Sperry JS . 2001. Functional and ecological xylem anatomy. Perspectives in Plant Ecology, Evolution and Systematics 4: 97–115.

[nph70926-bib-0062] Hansen J , Beck E . 1988. Evidence for ideal and non‐ideal equilibrium freezing of leaf water in frosthardy ivy (*Hedera helix*) and winter barley (*Hordeum vulgare*). Botanica Acta 101: 76–82.

[nph70926-bib-0063] Hoch G , Popp M , Körner C . 2002. Altitudinal increase of mobile carbon pools in *Pinus cembra* suggests sink limitation of growth at the Swiss treeline. Oikos 98: 361–374.

[nph70926-bib-0064] Hsiao TC , Acevedo E , Fereres E , Henderson DW . 1976. Water stress, growth and osmotic adjustment. Philosophical Transactions of the Royal Society of London. B, Biological Sciences 273: 479–500.

[nph70926-bib-0065] Hurvich CM , Tsai CL . 1995. Relative rates of convergence for efficient model selection criteria in linear regression. Biometrika 82: 418–425.

[nph70926-bib-0066] Imanishi HT , Suzuki T , Masuda K , Harada T . 1998. Accumulation of raffinose and stachyose in shoot apices of *Lonicera caerulea* L. during cold acclimation. Scientia Horticulturae 72: 255–263.

[nph70926-bib-0154] IPCC . 2012. In: Field CB , Barros V , Stocker TF , Qin D , Dokken DJ , Ebi KL , Mastrandrea MD , Mach KJ , Plattner GK , Allen SK *et al*., eds. Chapter 1: Climate change: New dimensions in disaster risk, exposure, vulnerability, and resilience. In: Managing the risks of extreme events and disasters to advance climate change adaptation. Cambridge, UK: Cambridge University Press, 582.

[nph70926-bib-0067] Klein G , Vitasse Y , Rixen C , Marty C , Rebetez M . 2016. Shorter snow cover duration since 1970 in the Swiss Alps due to earlier snowmelt more than to later snow onset. Climatic Change 139: 637–649.

[nph70926-bib-0068] Körner C . 2012. Treelines will be understood once the functional difference between a tree and a shrub is. Ambio 41: 197–206. 22864694 10.1007/s13280-012-0313-2 PMC3535059

[nph70926-bib-0069] Kramer PJ . 1940. Root resistance as a cause of decreased water absorption by plants at low temperatures. Plant Physiology 15: 63–79. 16653622 10.1104/pp.15.1.63 PMC438252

[nph70926-bib-0070] Lamacque L , Charrier G , Farnese FDS , Lemaire B , Améglio T , Herbette S . 2020. Drought‐induced mortality: branch diameter variation reveals a point of no recovery in lavender species. Plant Physiology 183: 1638–1649. 32404411 10.1104/pp.20.00165 PMC7401119

[nph70926-bib-0071] Lamacque L , Sabin F , Améglio T , Herbette S , Charrier G . 2022. Detection of acoustic events in lavender for measuring xylem vulnerability to embolism and cellular damage. Journal of Experimental Botany 73: 3699–3710. 35176148 10.1093/jxb/erac061

[nph70926-bib-0072] Lamhamedi M , Lambany G , Margolis H , Renaud M , Veilleux L , Bernier PY . 2001. Growth, physiology, and leachate losses in *Picea glauca* seedlings (1+ 0) grown in air‐slit containers under different irrigation regimes. Canadian Journal of Forest Research 31: 1968–1980.

[nph70926-bib-0073] Lanner RM . 2002. Why do trees live so long? Ageing Research Reviews 1: 653–671. 12362893 10.1016/s1568-1637(02)00025-9

[nph70926-bib-0074] Larcher W . 1963. Zur spätwinterlichen Erschwerung der Wasserbilanz von Holzpflanzen an der Waldgrenze. Berichte des Naturwissenschaftlich‐Medizinischen Vereins in Innsbruck 53: 125–137.

[nph70926-bib-0075] Larcher W . 1972. Der Wasserhaushalt immergrüner Pflanzen im Winter. Berichte der Deutschen Botanischen Gesellschaft 85: 315–327.

[nph70926-bib-0076] Larcher W , Mair B . 1968. Das Kälteresistenzverhalten von Quercus pubescens, Ostrya carpinifolia und Fraxinus ornus auf drei thermisch unterschiedlichen Standorten. Oecologia Plantarum 3: 255–270.

[nph70926-bib-0077] Lee CM , Thomashow MF . 2012. Photoperiodic regulation of the C‐repeat binding factor (CBF) cold acclimation pathway and freezing tolerance in Arabidopsis thaliana. Proceedings of the National Academy of Sciences, USA 109: 15054–15059. 10.1073/pnas.1211295109 PMC3443188 22927419

[nph70926-bib-0078] Levitt J , Levitt J . 1958. Frost, drought, and heat resistance. Vienna, Austria: Springer, 1–85.

[nph70926-bib-0079] Lintunen A , Lindfors L , Nikinmaa E , Hölttä T . 2017. Xylem diameter changes during osmotic stress, desiccation and freezing in *Pinus sylvestris* and *Populus tremula* . Tree Physiology 37: 491–500. 27998974 10.1093/treephys/tpw114

[nph70926-bib-0152] Lintunen A , Hölttä T , Kulmala M . 2013. Anatomical regulation of ice nucleation and cavitation helps trees to survive freezing and drought stress. Scientific Reports 3: 2031. 23778457 10.1038/srep02031 PMC3686780

[nph70926-bib-0080] Lintunen A , Paljakka T , Riikonen A , Lindén L , Lindfors L , Nikinmaa E , Hölttä T . 2015. Irreversible diameter change of wood segments correlates with other methods for estimating frost tolerance of living cells in freeze‐thaw experiment: a case study with seven urban tree species in Helsinki. Annals of Forest Science 72: 1089–1098.

[nph70926-bib-0081] Lloret F , Hurtado P , Espelta JM , Jaime L , Nikinmaa L , Lindner M , Martínez‐Vilalta J . 2024. ORF, an operational framework to measure resilience in social–ecological systems: the forest case study. Sustainability Science 19: 1579–1593.

[nph70926-bib-0082] Lloret F , Keeling EG , Sala A . 2011. Components of tree resilience: effects of successive low‐growth episodes in old ponderosa pine forests. Oikos 120: 1909–1920.

[nph70926-bib-0083] Lockhart JA . 1965. An analysis of irreversible plant cell elongation. Journal of Theoretical Biology 8: 264–275. 5876240 10.1016/0022-5193(65)90077-9

[nph70926-bib-0084] Mayr S , Améglio T . 2016. Freezing stress in tree xylem. Progress in Botany 77: 381–414.

[nph70926-bib-0085] Mayr S , Charra‐Vaskou K . 2007. Winter at the alpine timberline causes complex within‐tree patterns of water potential and embolism in *Picea abies* . Physiologia Plantarum 131: 131–139. 18251931 10.1111/j.1399-3054.2007.00942.x

[nph70926-bib-0086] Mayr S , Cochard H , Améglio T , Kikuta SB . 2007. Embolism formation during freezing in the wood of *Picea abies* . Plant Physiology 143: 60–67. 17041033 10.1104/pp.106.085704 PMC1761990

[nph70926-bib-0087] Mayr S , Gruber A , Bauer H . 2003. Repeated freeze–thaw cycles induce embolism in drought stressed conifers (Norway spruce, stone pine). Planta 217: 436–441. 14520570 10.1007/s00425-003-0997-4

[nph70926-bib-0088] Mayr S , Hacke U , Schmid P , Schwienbacher F , Gruber A . 2006a. Frost drought in conifers at the alpine timberline: xylem dysfunction and adaptations. Ecology 87: 3175–3185. 17249241 10.1890/0012-9658(2006)87[3175:fdicat]2.0.co;2

[nph70926-bib-0089] Mayr S , Schmid P , Laur J , Rosner S , Charra‐Vaskou K , Dämon B , Hacke UG . 2014. Uptake of water via branches helps timberline conifers refill embolized xylem in late winter. Plant Physiology 164: 1731–1740. 24521876 10.1104/pp.114.236646 PMC3982737

[nph70926-bib-0090] Mayr S , Schmid P , Rosner S . 2019. Winter embolism and recovery in the conifer shrub *Pinus mugo* L. Forests 10: 941.

[nph70926-bib-0091] Mayr S , Sperry JS . 2010. Freeze–thaw‐induced embolism in *Pinus contorta*: centrifuge experiments validate the ‘thaw‐expansion hypothesis’ but conflict with ultrasonic emission data. New Phytologist 185: 1016–1024. 20028475 10.1111/j.1469-8137.2009.03133.x

[nph70926-bib-0092] Mayr S , Wieser G , Bauer H . 2006b. Xylem temperatures during winter in conifers at the alpine timberline. Agricultural and Forest Meteorology 137: 81–88.

[nph70926-bib-0093] Mayr S , Wolfschwenger M , Bauer H . 2002. Winter‐drought induced embolism in Norway spruce (*Picea abies*) at the Alpine timberline. Physiologia Plantarum 115: 74–80. 12010469 10.1034/j.1399-3054.2002.1150108.x

[nph70926-bib-0094] McDowell N , Pockman WT , Allen CD , Breshears DD , Cobb N , Kolb T , Plaut J , Sperry J , West A , Williams DG *et al*. 2008. Mechanisms of plant survival and mortality during drought: why do some plants survive while others succumb to drought? New Phytologist 178: 719–739. 18422905 10.1111/j.1469-8137.2008.02436.x

[nph70926-bib-0095] McDowell NG . 2011. Mechanisms linking drought, hydraulics, carbon metabolism, and vegetation mortality. Plant Physiology 155: 1051–1059. 21239620 10.1104/pp.110.170704 PMC3046567

[nph70926-bib-0096] Meyer RF , Boyer JS . 1972. Sensitivity of cell division and cell elongation to low water potentials in soybean hypocotyls. Planta 108: 77–87. 24473747 10.1007/BF00386508

[nph70926-bib-0097] Michaelis P . 1934. Ökologische Studien an der alpinen Baumgrenze. V. Osmotischer Wert und Wassergehalt während des Winters in verschiedenen Höhenlagen. Jahrbucher fur Wissenschaftliche Botanik 80: 337–362.

[nph70926-bib-0098] Myking T , Solberg EJ , Austrheim G , Speed JD , Bøhler F , Astrup R , Eriksen R . 2013. Browsing of sallow (*Salix caprea* L.) and rowan (*Sorbus aucuparia* L.) in the context of life history strategies: a literature review. European Journal of Forest Research 132: 399–409.

[nph70926-bib-0099] O'Malley PE . 1979. Xylem sap flow and pressurisation in *Acer pseudoplatanus* L. Glasgow, UK: University of Glasgow.

[nph70926-bib-0100] O'Malley PER , Milburn JA . 1983. Freeze‐induced fluctuations in xylem sap pressure in *Acer pseudoplatanus* . Canadian Journal of Botany 61: 3100–3106.

[nph70926-bib-0101] Olson ME , Soriano D , Rosell JA , Anfodillo T , Donoghue MJ , Edwards EJ , León‐Gómez C , Dawson T , Camarero Martínez JJ , Castorena M *et al*. 2018. Plant height and hydraulic vulnerability to drought and cold. Proceedings of the National Academy of Sciences, USA 115: 7551–7556. 10.1073/pnas.1721728115 PMC6055177 29967148

[nph70926-bib-0102] Oogathoo S , Duchesne L , Houle D , Kneeshaw D , Bélanger N . 2024. Precipitation and relative humidity favours tree growth while air temperature and relative humidity respectively drive winter stem shrinkage and expansion. Frontiers in Forests and Global Change 7: 1368590.

[nph70926-bib-0103] Palacio S , Lenz A , Wipf S , Hoch G , Rixen C . 2015. Bud freezing resistance in alpine shrubs across snow depth gradients. Environmental and Experimental Botany 118: 95–101.

[nph70926-bib-0104] Palm E , Tveitereid M . 1979. On heat and mass flux through dry snow. Journal of Geophysical Research 84(C2): 745–749.

[nph70926-bib-0105] Peguero‐Pina JJ , Alquézar‐Alquézar JM , Mayr S , Cochard H , Gil‐Pelegrín E . 2011. Embolism induced by winter drought may be critical for the survival of *Pinus sylvestris* L. near its southern distribution limit. Annals of Forest Science 68: 565–574.

[nph70926-bib-0106] Pisek A , Larcher W . 1954. Relationship between drought resistance and frost hardiness in evergreens.

[nph70926-bib-0107] Pittermann J , Sperry J . 2003. Tracheid diameter is the key trait determining the extent of freezing‐induced embolism in conifers. Tree Physiology 23: 907–914. 14532014 10.1093/treephys/23.13.907

[nph70926-bib-0108] Pittermann J , Sperry JS . 2006. Analysis of freeze‐thaw embolism in conifers. The interaction between cavitation pressure and tracheid size. Plant Physiology 140: 374–382. 16377751 10.1104/pp.105.067900 PMC1326058

[nph70926-bib-0109] Power CC , Normand S , von Arx G , Elberling B , Corcoran D , Krog AB , Prendin AL . 2024. No effect of snow on shrub xylem traits: Insights from a snow‐manipulation experiment on Disko Island, Greenland. Science of the Total Environment 916: 169896. 38185160 10.1016/j.scitotenv.2024.169896

[nph70926-bib-0110] Rauscher HM , Isebrands JG , Host GE , Dickson RE , Dickmann DI , Crow TR , Michael DA . 1990. ECOPHYS: an ecophysiological growth process model for juvenile poplar. Tree Physiology 7(1–2–3‐4): 255–281. 14972923 10.1093/treephys/7.1-2-3-4.255

[nph70926-bib-0111] Reineke LH . 1932. A precision dendrometer. Journal of Forestry 30: 692–699.

[nph70926-bib-0112] Rixen C , Høye TT , Macek P , Aerts R , Alatalo JM , Anderson JT , Zong S . 2022. Winters are changing: snow effects on Arctic and alpine tundra ecosystems. Arctic Science 8: 572–608.

[nph70926-bib-0113] Saarinen T , Lundell R . 2010. Overwintering of *Vaccinium vitis‐idaea* in two sub‐Arctic microhabitats: a reciprocal transplantation experiment. Polar Research 29: 38–45.

[nph70926-bib-0114] Saarinen T , Rasmus S , Lundell R , Kauppinen OK , Hänninen H . 2016. Photosynthetic and phenological responses of dwarf shrubs to the depth and properties of snow. Oikos 125: 364–373.

[nph70926-bib-0115] Sakai A . 1966. Studies of frost hardiness in woody plants. II. Effect of temperature on hardening. Plant Physiology 41: 353–359. 16656262 10.1104/pp.41.2.353 PMC1086345

[nph70926-bib-0116] Sakai A , Larcher W . 1987. Low temperature and frost as environmental factors. In: Frost survival of plants: Responses and adaptation to freezing stress. Berlin, Heidelberg: Springer, 1–20.

[nph70926-bib-0117] Sakai A , Ötsuka K , Yoshida S . 1968. Mechanism of survival in plant cells at super‐low temperatures by rapid cooling and rewarming. Cryobiology 4: 165–173. 5746210 10.1016/s0011-2240(68)80110-5

[nph70926-bib-0118] Sakai A , Sakai S , Akiyama F . 1997. Do sprouting tree species on erosion‐prone sites carry large reserves of resources? Annals of Botany 79: 625–630.

[nph70926-bib-0119] Sala A , Piper F , Hoch G . 2010. Physiological mechanisms of drought‐induced tree mortality are far from being resolved. New Phytologist 186: 274–281. 20409184 10.1111/j.1469-8137.2009.03167.x

[nph70926-bib-0120] Sauter JJ . 1988. Temperature‐induced changes in starch and sugars in the stem of Populus × canadensis «robusta». Journal of Plant Physiology 132: 608–612.

[nph70926-bib-0121] Shakhmatov R , Hashiguchi S , Maximov TC , Sugimoto A . 2022. Effects of snow manipulation on larch trees in the taiga forest ecosystem in northeastern Siberia. Progress in Earth and Planetary Science 9: 1–16.

[nph70926-bib-0122] Simard S , Giovannelli A , Treydte K , Traversi ML , King GM , Frank D , Fonti P . 2013. Intra‐annual dynamics of non‐structural carbohydrates in the cambium of mature conifer trees reflects radial growth demands. Tree Physiology 33: 913–923. 24128848 10.1093/treephys/tpt075

[nph70926-bib-0123] Siminovitch D , Singh J , De La Roche IA . 1978. Freezing behavior of free protoplasts of winter rye. Cryobiology 15: 205–213. 668402 10.1016/0011-2240(78)90025-1

[nph70926-bib-0125] Sperry JS , Donnelly JR , Tyree MT . 1988. A method for measuring hydraulic conductivity and embolism in xylem. Plant, Cell & Environment 11: 35–40.

[nph70926-bib-0126] Sperry JS , Nichols KL , Sullivan JE , Eastlack SE . 1994. Xylem embolism in ring‐porous, diffuse‐porous, and coniferous trees of northern Utah and interior Alaska. Ecology 75: 1736–1752.

[nph70926-bib-0127] Sperry JS , Sullivan JE . 1992. Xylem embolism in response to freeze‐thaw cycles and water stress in ring‐porous, diffuse‐porous, and conifer species. Plant Physiology 100: 605–613. 16653035 10.1104/pp.100.2.605 PMC1075601

[nph70926-bib-0128] Sturm M , Holmgren J , König M , Morris K . 1997. The thermal conductivity of seasonal snow. Journal of Glaciology 43: 26–41.

[nph70926-bib-0129] Sulpice R , Pyl ET , Ishihara H , Trenkamp S , Steinfath M , Witucka‐Wall H , Gibon Y , Usadel B , Poree F , Piques MC *et al*. 2009. Starch as a major integrator in the regulation of plant growth. Proceedings of the National Academy of Sciences, USA 106: 10348–10353. 10.1073/pnas.0903478106 PMC2693182 19506259

[nph70926-bib-0130] Taulavuori K , Bauer E , Taulavuori E . 2011. Overwintering stress of *Vaccinium vitis‐idaea* in the absence of snow cover. Environmental and Experimental Botany 72: 397–403.

[nph70926-bib-0132] Tranquillini W . 1976. Water relations and alpine timberline. In: Water and plant life: problems and modern approaches. Berlin, Heidelberg, Germany: Springer Berlin Heidelberg, 473–491.

[nph70926-bib-0133] Tranquillini W . 1980. Winter desiccation as the cause for alpine timberline. NZFS FRI Technical Paper 70: 263–267.

[nph70926-bib-0134] Tranquillini W . 1982. Frost‐drought and its ecological significance. In: Physiological plant ecology II: water relations and carbon assimilation. Berlin, Heidelberg: Springer Berlin Heidelberg, 379–400.

[nph70926-bib-0135] Tyree MT , Cochard H . 1996. Summer and winter embolism in oak: impact on water relations.Annales Des Sciences Forestières 53: 173–180.

[nph70926-bib-0136] Urli M . 2013. Réponse des rabres forestiers aux changements globaux: approches biogéographique et écophysiologique (Doctoral dissertation, Université Sciences et Technologies‐Bordeaux I).

[nph70926-bib-0137] Vitasse Y , Bottero A , Cailleret M , Bigler C , Fonti P , Gessler A , Wohlgemuth T . 2019. Contrasting resistance and resilience to extreme drought and late spring frost in five major European tree species. Global Change Biology 25: 3781–3792. 31436853 10.1111/gcb.14803

[nph70926-bib-0138] Wheeler JA , Hoch G , Cortés AJ , Sedlacek J , Wipf S , Rixen C . 2014. Increased spring freezing vulnerability for alpine shrubs under early snowmelt. Oecologia 175: 219–229. 24435708 10.1007/s00442-013-2872-8

[nph70926-bib-0139] Wipf S , Rixen C . 2010. A review of snow manipulation experiments in Arctic and alpine tundra ecosystems. Polar Research 29: 95–109.

[nph70926-bib-0140] Zanne AE , Pearse WD , Cornwell WK , McGlinn DJ , Wright IJ , Uyeda JC . 2018. Functional biogeography of angiosperms: life at the extremes. New Phytologist 218: 1697–1709. 29603243 10.1111/nph.15114

[nph70926-bib-0141] Zhang MIN , Willison JHM , Cox MA , Hall SA . 1993. Measurement of heat injury in plant tissue by using electrical impedance analysis. Canadian Journal of Botany 71: 1605–1611.

[nph70926-bib-0142] Zhang T . 2005. Influence of the seasonal snow cover on the ground thermal regime: An overview. Reviews of Geophysics 43: RG4002.

[nph70926-bib-0144] Zweifel R , Häsler R . 2000. Frost‐induced reversible shrinkage of bark of mature subalpine conifers. Agricultural and Forest Meteorology 102: 213–222.

[nph70926-bib-0151] Zweifel R , Zimmermann L , Zeugin F , Newbery DM . 2006. Intra‐annual radial growth and water relations of trees: implications towards a growth mechanism. Journal of Experimental Botany 57: 1445–1459. 16556628 10.1093/jxb/erj125

