# Supplementary material for: Reduced snow cover at the alpine treeline: resistance and recovery of saplings
Source: New Phytol. 2026 Feb 3;250(3):1492–509. doi: 10.1111/nph.70926 (PMC13062728; doi:10.1111/nph.70926)
Supplement: Supplementary file 2 — Fig. S1 Stem diameter variations of one representative individual per species and per treatment (snow‐covered and snow‐free conditions) as well as mean stem and soil temperature and soil water potential during 12 months. Fig. S2 Correlation between visualized cellular mortality and spring loss of diameter of snow‐covered and snow‐free saplings of the five species. Please note: Wiley is not responsible for the content or functionality of any Supporting Information supplied by the authors. Any queries (other than missing material) should be directed to the New Phytologist Central Office. [file NPH-250-1492-s001.pdf]

**New Phytologist Supporting Information**

**Article title:**

# Reduced snow cover at the alpine treeline: resistance and recovery of saplings

**Authors:**

**Katline Charra-Vaskou, Guillaume Charrier, Andrea Ganthaler, Thierry Améglio and Stefan Mayr**

**Article acceptance date: 26 December 2025**

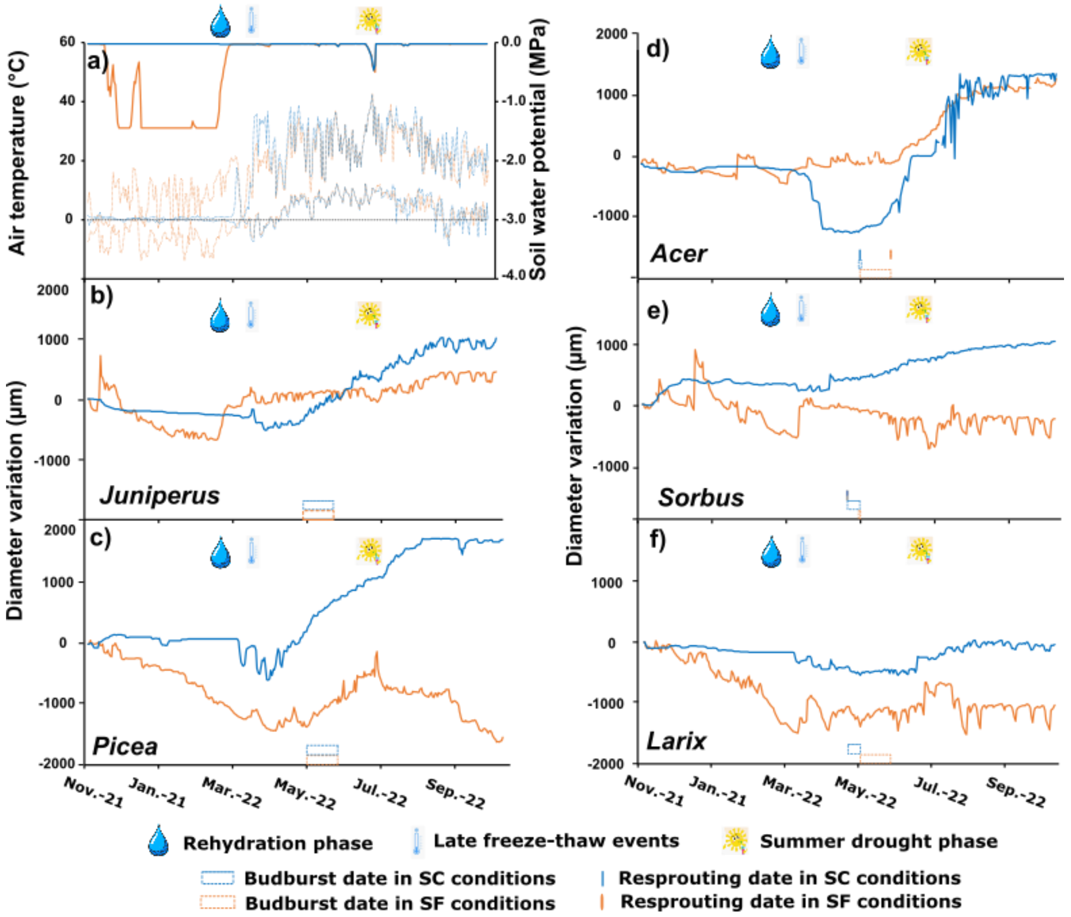


**Figure S1:** Stem diameter variations (µm) of one representative individual per treatment for *Juniperus* (box b), *Picea* (box c), *Acer* (box d), *Sorbus* (box e ) and *Larix* (box f) as well as mean stem and soil temperature and soil water potential during 12 months (September 2021 to September 2022, box a). Conditions in snow-covered (SC) plot are represented by blue lines and conditions in snow-free (SF) plot by orange lines.

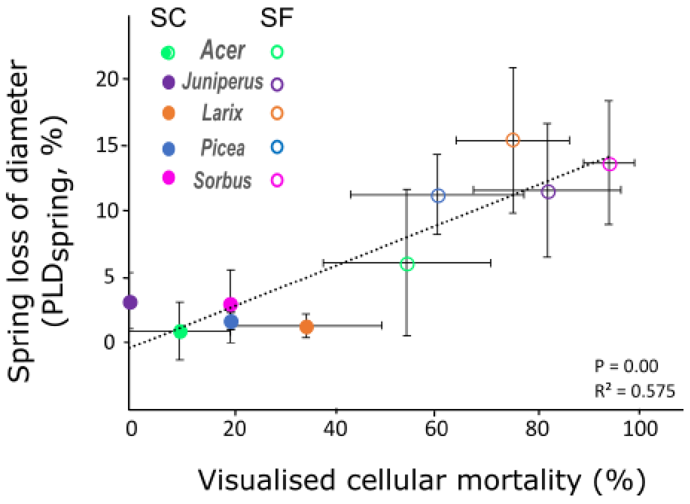


**Figure S2**: Percent visualised cellular mortality with percent spring loss of diameter ($PLD_{spring}$) of snow-covered (SC) and snow-free (SF) saplings of the five species. Living cell mortality was determined on short stem segments immersed 48h at room temperature in the TTC solution ( 0.5% (w/v) 2,3,5-Triphenyl-tetrazolium chloride) and then transferred to water. Within the following day cross section from the middle part of each sample were analysed with a light microscope to measure the percent of dead and unstained cells as TTC stains vital, metabolically active cells bright red due to a reduction of the compound to water insoluble formazan, while all other cells remain unstained (from *Ganthaler et al., 2022*). The dotted line indicates the best linear regression.
